# Effect of paratuberculosis vaccination before and after oral experimental infection with *Mycobacterium avium* subspecies *paratuberculosis* in goats

**DOI:** 10.1080/01652176.2025.2566363

**Published:** 2025-10-01

**Authors:** Marcos Royo, Natalia Elguezábal, Rakel Arrazuria, Julio Benavides, Miguel Fernández

**Affiliations:** a Departamento de Sanidad Animal, Facultad de Veterinaria, Campus de Vegazana, Universidad de León, León, España; b Departamento de Sanidad Animal, Instituto de Ganadería de Montaña (CSIC-Universidad de León), Grulleros, España; c Animal Health Department, NEIKER-Instituto Vasco de Investigación y Desarrollo Agrario, Basque Research and Technology Alliance (BRTA), Derio, Bizkaia, Basque Country, Spain

**Keywords:** Paratuberculosis, vaccination, *Mycobacterium avium* subsp *paratuberculosis*, immune response, lesion, granuloma, goats, ruminants

## Abstract

Vaccination against paratuberculosis, before or after infection with *Mycobacterium avium* subsp. *paratuberculosis* (Map), could affect the progression of paratuberculosis, the development of lesions, the peripheral and local immune response, or the colonization of Map in tissues and its elimination through feces. An experimental study was conducted with thirty-five 1.5-month-old kids, which were separated into 6 experimental groups that include different intervention combinations (vaccinated, non-vaccinated, challenged and non-challenged) at different points and slaughtered at 120 and 330 days post-infection. The use of an inactivated vaccine against paratuberculosis could avoid clinical disease manifestation but does not prevent the tissue colonization, even when applied before Map exposure, achieving a reduction in the presence of viable bacteria in tissues and limiting the progression toward diffuse lesions. The therapeutic effect in vaccinated animals could not be confirmed. In this sense, vaccination not only modulates the immune response in terms of the production of IFN-γ and antibodies in peripheral blood and reduces tissue damage but also contributes to limiting the spread of infection through reduced bacterial shedding especially in goats vaccinated before Map infection.

## 1. Introduction

Johne’s disease or paratuberculosis is a chronic infectious disease caused by *Mycobacterium avium* subspecies *paratuberculosis* (Map), affecting domestic and wild ruminants worldwide (Chiodini et al. 1984, Groenendaal et al. 2015). The disease causes serious economic losses in the ruminant livestock industry linked to the decreased productivity on affected farms (Kennedy and Benedictus 2001; Garcia and Shalloo, 2015). The proposed link between Map and human Crohn’s disease is controversial (Autschbach et al. 2005; Parrish et al. 2009) and has increased interest in this pathogen (Chiodini 1989; Bentley et al. 2008; Naser et al. 2014; Oken et al. 2017).

Map infection usually occurs during the first weeks of life, mainly through the fecal-oral route (Clarke 1997; Barkema et al. 2009), although clinical signs generally appear in adulthood, marked by progressive weight loss that can be accompanied by diarrhea associated with diffuse granulomatous enteritis (Chiodini et al. 1984; Dennis et al. 2011). However, not all the infected animals develop overt disease. In fact, most remain subclinical throughout their lives (Whitlock and Buergelt 1996), exhibiting latent, limited, focal granulomatous lesions in intestinal lymphoid tissue, which are considered forms of resistance in sheep (Fernández et al. 2014). The mechanisms behind these varied responses remain unclear, but the immune response in sheep and cows is suspected to play a key role in disease progression (Pérez et al. 1996, 1997; Vázquez et al. 2013; Fernández et al. 2014, 2017). According to this, studying lesion types after Map infection has proven to be a valuable indicator of disease progression, linking different lesions to latent, subclinical or clinical stages (Pérez et al. 1997; Delgado et al. 2013; Vázquez et al. 2013; Fernández et al. 2014, 2017, 2022). Moreover, a relationship between clinical phases and heightened humoral immune responses in domestic ruminants, such as cattle, sheep, and goats has been established (Pérez et al. 1997, 1999; Vazquez et al. 2013; Fernández et al. 2014; Stefanova et al. 2023). Current hygienic and test-cull strategies have proven inadequate for controlling paratuberculosis, whereas vaccination is seen as the most cost-effective tool for small ruminants (Bastida and Juste 2011; Stefanova et al. 2023). While vaccination does not prevent infection, it significantly reinforces the specific immune response, reduces clinical signs, economic losses, and Map shedding (Reddacliff et al. 2006; Windsor 2006; Bastida and Juste 2011, Juste et al. 2021; Bannatine et al. 2022; Stefanova et al. 2023). In fact, vaccination is recommended early in life to generate a protective effect in vaccinated animals against infection with Map, which predominantly occurs in this period (Juste et al. 1994; Sweeney et al. 2009; Tewari et al. 2014; Agulló-Ros et al. 2022). Several studies have shown that vaccinating adult sheep, goats and cows, likely already exposed to or infected with Map, can reduce the incidence of new clinical cases (Pérez et al. 1995; Corpa et al. 2000a; García-Pariente et al. 2003; Alonso-Hearn et al. 2012; Santema et al. 2013; Fernández et al. 2022). No previous studies in controlled conditions have confirmed this possibility, except for Gwozdz et al. (2000b), who evaluated the effect of vaccination 2 weeks after infecting lambs. However, the establishment of the previous infection was not proven. Arrazuria et al. (2016) conducted a similar study in rabbits and found that vaccination after infection had a slightly stronger protective effect than vaccination before infection, suggesting a therapeutic effect in this model. Further studies to test this hypothesis under controlled conditions in ruminants, using a commercial vaccine commonly employed have not been developed and are needed since the applicability to field conditions would be interesting. For all these reasons, an experimental goat model was employed to assess the effect of vaccination, before and after experimental infection with Map, on the pathogenesis of paratuberculosis, evaluating the development of lesions, the peripheral and local immune response, and the colonization of Map in tissues and its shedding through feces.

## 2. Material and methods

### 2.1. Ethical approval

This study was conducted under the regulations and corresponding authorization for experimental procedures with animals according to the Spanish and European legislation (Law 32/2007, RD 53/2013 and Directive 2010/63/UE) with the approval of the Ethics Committee (CEE-IGM2017-001) and the final authorization of the competent authority (Junta de Castilla y León).

### 2.2. Animals and experimental design

Thirty-five 1.5 month-old male goat kids of Murciano-Granadina breed that came from a herd of dairy goats officially confirmed as free of tuberculosis and without notice of clinical signs related to paratuberculosis for at least the previous 5 years were used in this experiment. Prior to the beginning of the study, all female goats of the herd tested negative for exposure to Map by IFN-γ release assay (IGRA) and indirect ELISA (IDVet^®^). Clinically healthy goat kids, aged between 1 to 1.5 months, were housed in the animal facilities of the Instituto de Ganadería de Montaña (IGM CSIC-ULE).

Goats were previously acclimated, kept in separate areas under the same housing and feeding conditions, and divided randomly in six different groups based on vaccination and infection status (Table 1):

**Table 1.** Distribution of the animals in the different experimental groups and the interventions according to the moment in which they were vaccinated, challenged or slaughtered.

Groups	Nr of animals (total)	Interventions conducted with animals from each group during the study					
		Vaccinated 30 days before infection (−30 dpi)	30 days post vaccination: map infection (0 dpi)	Comparative intradermal skin test at 90 dpi	Culling and sampling at 120 dpi	Vaccinated at 150 dpi	Culling and sampling at 330 dpi
VI	8	8	8		3	–	4^A^
VNI	4	4	–		2	–	1^B^
NVI	9	–	9		5^C^	–	3
IV	5	–	5		–	5	5
NIV	2	–	–		–	2	2
NVNI	7	–	–		2	–	3^D^
Total	35	12	22		12	7	18

^A^ One kid from this group was euthanized at 245 dpi.

^B^ One kid from this group was withdrawn from the study at 186 dpi.

^C^ One kid from this group was withdrawn from the study at 96 dpi.

^D^ Two kids from this group were withdrawn from the study at 238 dpi and 302 dpi. Nr: number of animals.

dpi: days post-infection.

- VI (Vaccinated-Infected): 8 goat kids vaccinated 30 days before experimental infection (−30 dpi) and subsequently infected at day 0 (30 dpv).
- VNI (Vaccinated-Not Infected): 4 goat kids vaccinated 30 days before experimental infection but not infected.
- NVI (Not Vaccinated-Infected): 9 goat kids not vaccinated and experimentally infected at 0 dpi.
- IV (Infected-Vaccinated): 5 goat kids infected at 0 dpi and vaccinated at 150 dpi.
- NIV (Not Infected-Vaccinated): 2 goat kids not infected but vaccinated at 150 dpi.
- NVNI (Not Vaccinated-Not Infected): 7 goat kids neither vaccinated nor infected (negative control group).

### 2.3. Clinical signs assessment

Animals belonging to the experiment were under continuous health monitoring supervision performed by the veterinarians and members of the research group. General clinical signs such as apathy, fever, prolonged or abnormal recumbency were evaluated when they appeared, and specifically those symptoms of intestinal disease such as diarrhea, loss of appetite, weight loss and emaciation, were recorded in the animal research project tracking book.

### 2.4. Vaccination and infection challenge

**Figure 1.**
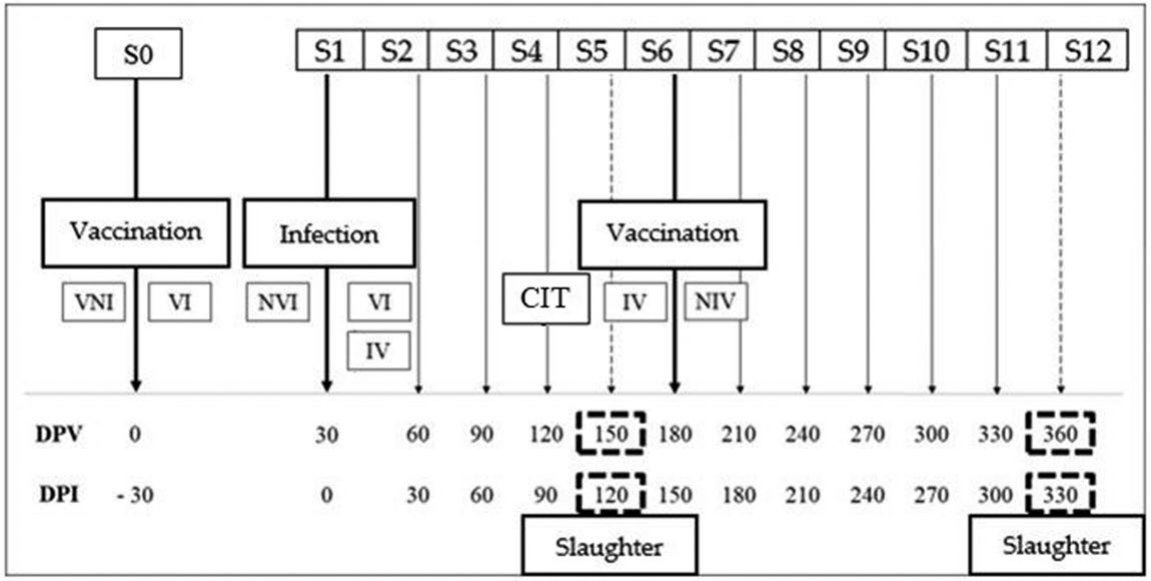
Scheme of experimental design. S: sampling; DPV: days post-vaccination; DPI: days post-infection; VNI: vaccinated at −30 dpi and not infected; VI: vaccinated at −30 dpi and infected; NVI: not vaccinated, but infected; IV: infected and vaccinated at 150 dpi; NIV: not infected but vaccinated at 150 dpi. NVNI: non-vaccinated and non-infected, slaughtered at 120 and 330 dpi. (not shown). CIT: cervical intradermal skin test. S: sampling point number.

The experimental scheme including interventions and timepoints is detailed in Figure 1. Immunization was performed with Silirum^®^ vaccine (CZ Veterinaria, Porriño, Pontevedra, ES), registered in cattle and composed of the heat-inactivated 316 F Map strain in a high-refinement mineral oil adjuvant. Animals were vaccinated according to the experimental design with 1 ml of the vaccine subcutaneously, with one dose per animal on the left side of the kid’s back area, as recommended by the manufacturer.

According to the experimental design, animals belonging to the infected groups were orally challenged with the K-10 Map strain (NEIKER, Derio, ES), bovine or type C strain, a reference strain, whose complete genome has been sequenced and which lacks certain virulence factors found in field strains, prepared as described previously (Arrazuria et al. 2015). Inoculum was administered orally, using an esophageal probe attached to an automatic syringe (Haupner, GE) with a total dose of 1.2 × 10^10^ cfu of Map. This total amount was divided into 4 doses of 10 ml each with a concentration of 0.3 × 10^10^ mycobacteria per animal suspended in 10 ml of PBS (phosphate-buffered saline), given every 3 days, considering the day of infection as the first inoculum administered (day 0). At 120 dpi, a first slaughter was performed to check the infection status and the development of associated lesions (Table 1). Subsequently, at 150 dpi, two groups (IV and NIV) were vaccinated subcutaneously with the same heat-inactivated vaccine Silirum^®^ (CZ Veterinaria, Porriño, ES). At 330 dpi (360 dpv), all the survivor goats were slaughtered. The remaining goats served as neither vaccinated nor infected controls. Goats were euthanized with 0.1 mg/kg of intramuscular xylazine (Rompun^®^; Bayer Animal Health, Mannheim, DE) and 0.1 ml/kg of T61 (MSD Animal Health, Salamanca, Spain) administered intravenously followed by exsanguination.

### 2.5. Peripheral immune response determination

#### 2.5.1. Cellular immune response – interferon-γ release assay (IGRA)

**Figure 2.**
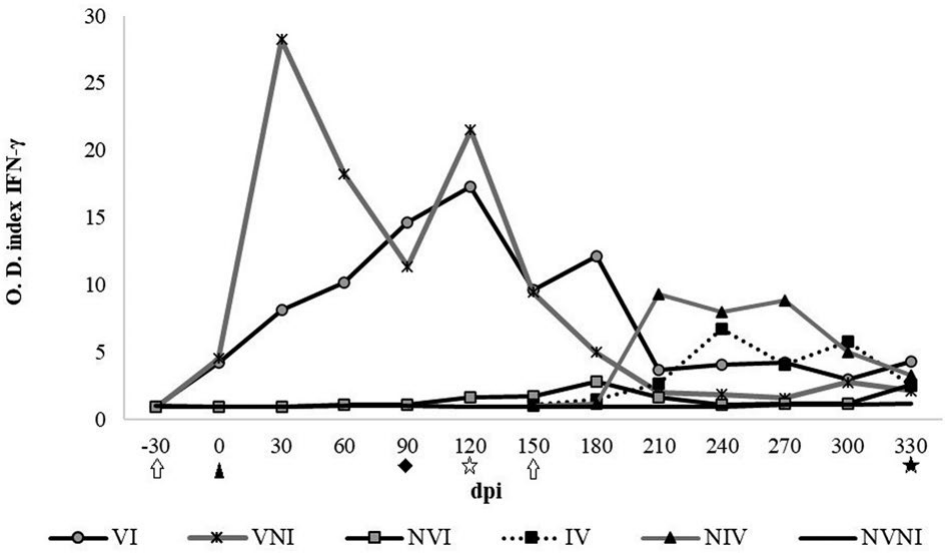

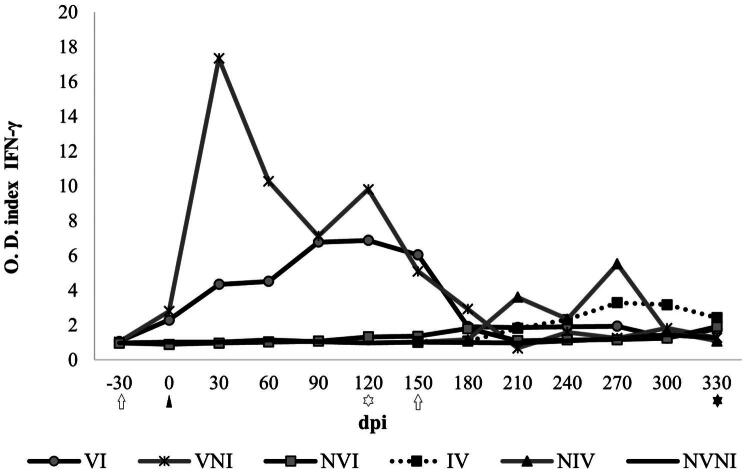
(a) Kinetics of IFN-γ release after stimulation with avian PPD. VI: vaccinated at −30 dpi and infected (arithmetic mean ± SD); VNI: vaccinated at – 30 dpi and not infected; NVI: not vaccinated and infected; NVNI: not vaccinated and not infected; IV: infected and vaccinated at 150 dpi (arithmetic mean ± SD); NIV: not infected and vaccinated at 150 dpi. O.D.: Optical density quotient (y axis); dpi: days post-infection (x axis). White arrow: Vaccination (30 days prior to infection and 150 dpi; black arrowhead: infection (0 dpi); black rhombus: cervical intradermal skin test (90 dpi); white star: first slaughter (120 dpi); black star: second slaughter (330 dpi). IFN-γ was significantly higher in animals in groups VI and VNI than in those in groups NVI and NVNI between 0 and 180 dpi (*p* < 0.05). (b) Kinetics of specific IFN-γ release after stimulation with bovine PPD. VI: vaccinated at −30 dpi and infected (arithmetic mean ± SD); VNI: vaccinated at – 30 dpi and not infected; NVI: not vaccinated and infected; NVNI: not vaccinated and not infected; IV: infected and vaccinated at 150 dpi (arithmetic mean ± SD); NIV: not infected and vaccinated at 150 dpi. O.D.: Optical density quotient (*y* axis); dpi: days post-infection (*x* axis). White arrow: Vaccination (30 days prior to infection and 150 dpi; black arrowhead: infection (0 dpi); black rhombus: cervical intradermal skin test (90 dpi); white star: first slaughter (120 dpi); black star: second slaughter (330 dpi).

Blood samples were collected monthly (−30, 0, 30, 60, 90, 120, 150, 180, 210, 240, 270 and 300 dpi) from the jugular vein into 10 ml heparinized Vacutainer^®^ tubes (Becton Dickinson, Plymouth, UK) to assay the IFN-γ release (Figure 1). Within 4 hours after collection, three separate 1.5 ml aliquots from every blood sample were mixed with 100 µl (20 μg/ml) of avian PPD (CZ Veterinaria, Porriño, ES), 100 µl (20 μg/ml) of bovine PPD (CZ Veterinaria, Porriño, Spain) and 100 µl of PBS (Phosphate-Buffered Saline), respectively. 20 μl of concanavalin A (5 μg/ml) (Sigma-Aldrich, Lyon, France) was used as a positive stimulation control. Incubation took place in a 5% CO2 atmosphere incubator for 48 h at 37 °C. The plates were then centrifuged for 10 min at 3700 rpm, the plasma supernatant was collected from each well and transferred to ‘Deep-Well’ plates (Thermo Fisher Scientific, Illkirch, France), where they were stored at −20 °C until the IFN-γ capture ELISA BOVIGAM^®^ (TB Kit, Thermo Fisher Scientific, Waltham, USA) was employed to assay the IFN-γ release following the manufacturer’s instructions. Every stimulated sample was evaluated in duplicate. The absorbance values were measured spectrophotometrically using an ELX800 ELISA reader (Bio-Tek Instruments, Winooski, USA) at 450 nm. The results were expressed as the optical density quotient (OD index in the *y* axis in the graphs) between the mean optical densities (OD) of each sample stimulated with the different antigens divided by the OD of the same sample stimulated with PBS (Figure 2a and 2b).

#### 2.5.2. Comparative intradermal skin test (SICCT)

At 90 dpi, all goats were intradermally injected in the neck area with 0.1 ml of johnin (55 μg/ml) on the left side (supplied by NEIKER, Derio, Spain), and 0.1 ml of bovine and avian PPDs (20 μg/ml each) (CZ Veterinaria, Porriño, ES) on the right side, separated by at least 10 cm. The skin fold thickness was measured before inoculation and 72 h later (RD 2611/1996). The results were evaluated with a millimeter caliper and were expressed as an increase in skin thickness. NVI and IV as well as NVNI and NIV were considered together.

#### 2.5.3. Indirect ELISA

Sera obtained by centrifugation of whole blood samples without anticoagulant were used to assess the peripheral production of antibodies against Map. An indirect ELISA was performed (ID Vet^®^), following the manufacturer’s instructions. The absorbance values were measured spectrophotometrically at 405 nm using an ELX800 ELISA reader (Bio-Tek Instruments, Winooski, USA). A modified read-out was applied for the ELISA. The results were expressed as a quotient (OD ratio, in the y axis in the graphs) between the OD of each sample and the OD of positive control, multiplied by 1000. Sample with a ratio > 400 was considered positive.

#### 2.5.4. Characterization of lymphocyte subpopulations in peripheral blood

One week before each slaughter, peripheral blood mononuclear cells (PBMCs) were recovered from 20 ml of whole blood from Vacutainer^®^ heparinized tubes. Blood samples were mixed 1:1 with PBS and the mix was centrifuged at room temperature for 20 min at 1800 rpm. The leukocyte layer was carefully collected and added over 7 ml of Lymphoprep^®^ (Stemcells Technologies, Canada). The lymphoprep-leukocyte preparation was centrifuged for 30 min at 2100 rpm, and the layer was collected and washed three times with HBSS (Hanks Balanced Salt Solution) at 4 °C for 10 min at 1100 rpm. Finally, PBMCs were re-suspended at a final concentration of 2 × 10^6^ cells/ml in 9 ml of RPMI (Axis-Shield, Oslo, Norway) supplemented with antibiotics (10,000 U/ml penicillin, 10 mg/ml streptomycin and 25 µg/ml amphotericin B) and 1 ml of fetal bovine serum (Gibco^®^, Invitrogen^TM^, EEUU).

PBMCs were labeled with 17D1 (anti-CD4), MCA2216GA (anti-CD8), MCA838G (anti-WC1) and MCA1195 (anti-CD21) monoclonal primary antibodies (AbD Serotec, UK) by incubation for 1 h at 37 °C. Unbound antibodies were discarded by washing 3 times with PBS. After, incubation with polyclonal FITC-conjugated secondary antibody (Dako Agilent, Santa Clara, USA) cell suspensions were washed to remove unbound secondary antibody, and these were finally resuspended in PBS. Finally, samples were stored at 4 °C in darkness until prompt analysis in a CyAn ADP (Beckman Coulter) with Summit Software (Dako, Denmark) and Flowing Software 2.5.1 (University of Turku, Finland).

### 2.6. Pathological studies

Complete necropsies were performed in all animals of the study. Gross examination was done focusing on the gut and related lymph nodes. Histological samples were collected from ileocecal valve (ICV), ileum (distal, mid, and proximal), jejunum (distal, mid, and proximal) and Peyer’s patches (JPP) from distal, medium, and proximal zones of jejunum. Three different lymph nodes were considered: caudal jejunal lymph node (CJLN), mid jejunal lymph node (MJLN), and ileocecal lymph node (ICLN). Additionally, tissue samples from the vaccine injection site and the associated prescapular lymph node were taken for histopathological examination. All tissue samples were fixed in 10% neutral buffered formalin and were processed following the histopathological routine. Sections were stained with hematoxylin and eosin (HE), Ziehl-Neelsen (ZN) to detect acid-fast bacilli (AFB) and Masson’s Trichrome to visualize connective tissue.

Lesions compatible with Map infection were classified following the guidelines previously proposed (Pérez et al. 1996; Delgado et al. 2013; Fernández et al. 2014), based on the form, distribution, and location of granulomas: *focal* lesion characterized by granulomas within lymphoid tissue of the mucosa; *multifocal* forms when the granulomas spread to the related lamina propria (multifocal a) or not related (multifocal b); and *diffuse* lesions involving large areas of disrupted mucosa. Focal and multifocal forms are considered associated with subclinical stages of the disease, whereas diffuse lesions with clinical signs.

To assess the intensity of the lesions and compare the results between the experimental groups, a quantitative assessment was performed in the 10 intestinal and 3 lymph node tissue sections by counting granulomas. Three randomly selected fields from each intestinal location and lymph nodes were used for granuloma counting. The average number of granulomas counted per section was then calculated. In areas with Peyer’s patches, a distinction was made between areas with lymphoid tissue and the adjacent lamina propria. Lesions were evaluated and counted using a Nikon Eclipse 2000 microscope and using Image J software (Rasband, WS, ImageJ, US National Institutes of Health, Bethesda, Maryland, USA).

### 2.7. Microbiological studies

#### 2.7.1. Bacterial culture

Fecal samples were taken at samplings S0 (- 30 dpi), S5 (120 dpi) and S12 (330 dpi) (Figure 1). In addition, during slaughter, tissue samples (ileocecal valve, distal ileum, mid jejunal Peyer’s patch, and caudal jejunal lymph node) were collected. Due to a shortage of ileocecal valve, this tissue was mixed and cultured with distal ileum. All samples underwent decontamination following Adúriz et al. (1995). Four drops (150 μl)/per tube of the decontaminated suspension were seeded on solid media. Each sample was placed on two Middlebrook 7H9 medium tubes (Difco, USA) supplemented with OADC (Difco Laboratories, USA) and two Herrold’s egg yolk medium (HEYM) tubes (Difco, USA). Both media were supplemented with mycobactin J (Allied Monitor, Fayette, USA), penicillin (Difco Laboratories, USA), chloramphenicol (Sigma-Aldrich, Madrid, Spain) and amphotericin B (Sigma-Aldrich, Madrid, Spain). Seeded tubes were incubated at 37 °C and monitored monthly for growth for up to one year. Samples were termed as positive if characteristic colonies were observed in at least one of the duplicates and, finally, these colonies were confirmed to be Map by IS900 PCR and Ziehl-Neelsen staining.

#### 2.7.2. Nested PCR

Map detection in paraffin-embedded tissues was assessed by a nested PCR. A total of 10 µm thick tissue sections of the ileocecal valve, mid jejunal Peyer’s patches, and caudal jejunal lymph node was used for DNA extraction. DNA was isolated using Speedtools Tissue (Biotools^®^, B&M labs SA, Madrid, ES) following manufacturer’s instructions. Isolated DNA was stored at – 20 °C until subsequent analysis.

Nested PCR consisted of two consecutive PCR; the second round was performed to re-amplify the template obtained in the first round. First round was performed using primers and conditions previously described (Garrido et al. 2000). The second round was performed following instructions previously described (Delgado et al. 2012), ending in an amplified fragment of 226 pb. The amplified products were analyzed by electrophoresis on a 2% agarose gel stained with GelRed (Biotium, Fremont, USA) and visualization under ultraviolet light (GelDoc XR+, BioRad, USA).

### 2.8. Local immune response

#### 2.8.1. Isolation of mononuclear leukocytes from tissues

Tissue samples (prescapular lymph node, caudal jejunal lymph node, mid jejunal lymph node, mid jejunal Peyer’s patch, and distal ileum) from slaughtered animals were collected in 10 ml of RPMI and processed in the laboratory shortly after collection. Jejunum and ileum pieces were longitudinally opened showing the mucosa and washed with PBS until fecal remains were eliminated, whereas the fat adjacent to lymph nodes and the excess tissue around each Peyer Patch was removed and sliced in little pieces using a scalpel blade. Peyer’s patches were scraped and minced, and the resultant mucosa was immersed in RPMI. Minced tissue was suspended in 11 ml of PBS with EDTA (2 mM), homogenized and processed with a stomacher blender (Seward Stomacher^®^, Worthing, United Kingdom) for 2.5 min and then 10 ml from the upper homogenized portion was passes through 40 µm filter (Thermo Fischer Scientific, Madrid, Spain). The resultant mixture was carefully transferred into 10 ml of Lymphoprep^®^. The Lymphoprep-tissue preparation was centrifuged at room temperature for 30 min at 2100 rpm. The mononuclear leukocyte layer was aspirated, washed three times with HBSS at 4 °C for 10 min at 1100 rpm, counted in a Neubauer chamber and resuspended in 9 ml of RPMI supplemented with antibiotics (10,000 U/ml penicillin, 10 mg/ml streptomycin and 25 µg/ml amphotericin B) and 1 ml of fetal bovine serum (Gibco^®^, InvitrogenTM, EEUU) at a final concentration of 2 × 10^6^ cells/ml. Cell viability determined by trypan blue dye exclusion was above 80% for mononuclear lymphocytes.

#### 2.8.2. Characterization of lymphocyte subpopulations in tissues

Mononuclear leukocytes were labeled with the same panel of monoclonal primary antibodies used for PBMCs, following the same protocol mentioned before.

#### 2.8.3. *In vitro* IFN-γ secretion

500 µl of the cell suspension from tissues was added to 3 × 3 wells of 48-well plates (Corning Incorporated, Corning, USA). Every sample was incubated in triplicate with 30 µl (20 μg/ml) of avian PPD (CZ Veterinaria, Porriño, ES), 30 µl (5 μg/ml) of concanavalin A (as positive control) and 20 µl of PBS (as negative control). Plates were incubated in a humidified atmosphere of 5% CO_2_ at 37 °C for 48 h. After that phase, plates were centrifuged for 10 min at 3700 rpm and the resultant supernatant was collected and stored at −20 °C until interferon-γ quantification was performed with the commercial kit BOVIGAM^®^ (Thermo Fisher Scientific, USA). Results were expressed as the quotient between the OD of avian PPD-stimulated sample and the OD of the same sample incubated with PBS.

### 2.9. Statistical analysis

The data from the IFN-γ and ELISA indices, the increase in skin thickness in the IDTB, as well as the granuloma count and values of the different lymphocyte subpopulations, were subjected to an analysis of variance using the GLM (General Linear Model) method from the SAS statistical software package (SAS Institute Inc., Cary, NC, USA, 9.1). Prior to this, a logarithmic transformation of the results of the IFN-γ indices, ELISA, and granuloma count was carried out to ensure normal distribution. Although non-parametric tests are a good alternative when data do not meet the normality assumption, parametric tests are more powerful and provide more precise and reliable results, particularly with small sample sizes or high variability of the size of experimental groups. For the same purpose, the data from the lymphocyte subpopulations, obtained as percentages, underwent a square root arcsine transformation of the proportion. The differences between each experimental group at each sampling were evaluated using a Student’s t-test for adjusted means, employing the Tukey-Kramer correction for multiple comparisons. A statistical significance threshold of 0.05 was considered. The data were analyzed using the SAS software (SAS Institute Inc., Cary, NC, USA).

## 3. Results

### 3.1. Clinical signs assessment

No specific clinical signs related to paratuberculosis such as diarrhea, loss of appetite, weight loss or emaciation, were detected in any of the animals after monitoring throughout the study.

It is worth noting that five kids from different groups (VI, IV, VNI, NVI and NVNI) were euthanized at different times due to the appearance of general clinical signs not specifically associated with paratuberculosis (apathy, prolonged recumbency and difficulty urinating). Despite this, a complete necropsy was performed to determine the cause of death (urolithiasis in most cases), but the samples were not considered for the experiment.

### 3.2. Peripheral immune response determination

#### 3.2.1. Cellular immune response – interferon-γ release assay (IGRA)

The release of IFN-γ varied among the experimental groups when whole blood was incubated with avian purified protein derivative (PPD). A significant increase in IFN-γ release (*p* < 0.05) was observed in the vaccinated groups, VI and VNI compared to the non-vaccinated groups NVI and NVNI). This difference persisted from 0 dpi (30 dpv) to 180 dpi (210 dpv) in response to avian PPD stimulation. Although no significant differences were found between the two vaccinated groups, the VNI group exhibited higher net IFN-γ values at all time-points except at 90 dpi compared to group VI at this point. The VNI group reached peak levels of IFN-γ at 30 dpi (60 dpv), while group VI peaked later at 120 dpi (150 dpv). A significant increase in the IFN-γ index (*p* < 0.05) was also noted for both groups vaccinated at 150 dpi (IV and NIV) compared to the non-vaccinated groups (NVI and NVNI), during the interval between 210 dpi (60 dpv) and 300 dpi (150 dpv). The highest peaks were observed at 210 dpi (60 dpv) in group NIV and at 240 dpi (90 dpv) in group IV (Figure 2a). The non-vaccinated but infected group (NVI) exhibited a similar behavior to that of the control group up to 120 dpi. After this period, the release of interferon became significantly different (*p* < 0.05) to 210 dpi, after which no further IFN-γ differences were observed until the final sampling and slaughter at 330 dpi (Figure 2a).

The kinetics of IFN-γ release in response to stimulation with bovine PPD followed a similar pattern to that observed with avian PPD. Between 0 and 180 dpi, the vaccinated groups (VI and VNI) demonstrated significantly higher IFN-γ release compared to the non-vaccinated groups (NVI and NVNI). Consistent with previous observations related to avian PPD, the IFN-γ indexes were higher in group VNI than in group VI, but only at 30 dpi (*p* < 0.05). Both IV and NIV groups showed increased production of this cytokine (*p* < 0.05) at 150 dpi and between 210 dpi (60 dpv) and 270 dpi (120 dpv), compared to the unvaccinated groups (NVI and NVNI). In the NVI group, no differences were observed in the mean IFN-γ index after stimulation with bovine PPD compared to the NVNI group throughout the entire experiment., There were also no significant differences in IFN-γ production after stimulation with bovine PPD between animals in the vaccinated groups (VI and VNI) and those in the unvaccinated groups (NVI and NVNI) most of the time. However, significant differences were noted between these groups between 90 dpi (during SICCT) and 120 dpi (*p* < 0.05) (Figure 2b).

#### 3.2.2. Cellular immune response – comparative intradermal skin test (SICCT)

The SICCT was carried out at 90 dpi (120 dpv), timepoint at which the 150-dpi vaccination had not yet been performed. Vaccinated groups (VI and VNI) had a significantly higher increase (*p* < 0.05) compared to the not vaccinated groups (NVI-IV and NVNI-NIV) at the SICCT moment for PPDs at 72 h after inoculation. NVI group showed significant differences compared to the NVNI-NIV (*p* < 0.05) only with avian PPD.

**Figure 3.**
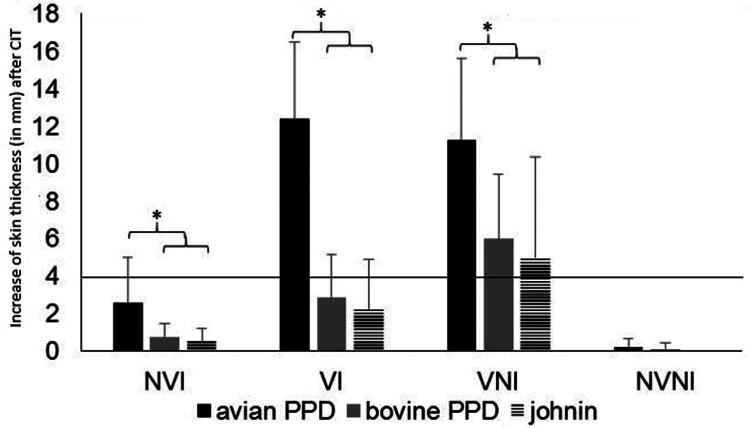
Mean increase of skin thickness, in millimeters (mm; *y* axis), from different experimental groups at 90 dpi after the cervical intradermal skin test with avian PPD, bovine PPD or johnin. NVI: not vaccinated and infected (included IV-infected and vaccinated, prior to vaccination); VI: vaccinated at −30 dpi and infected; VNI: vaccinated at – 30 dpi and not infected; NVNI: not vaccinated and not infected (included NIV-non-infected but vaccinated prior to vaccination). *significantly different (*p* < 0.05).

When comparing the increase in skin thickness depending on the antigen used, in the three treated groups (NVI-IV, VI, VNI), it was significantly higher (*p* < 0.05) for avian PPD compared to bovine or johnin PPD, among which there were no differences (Figure 3).

If these results are analyzed with the official criteria for the tuberculosis, in the case of comparative, no animal was found to be tuberculosis positive in any of the groups vaccinated or infected with Map. However, when the simple intradermal skin test (SICT) criteria were applied, (positive if the increase in skin thickness is 4 mm or above) there were 7 positive kids in the VNI and VI groups (58.3%). Among these, 3 presented an increase in thickness of 4 mm, two of 7 mm and one of 9 mm (VI).

#### 3.2.3. Humoral immune response

**Figure 4.**
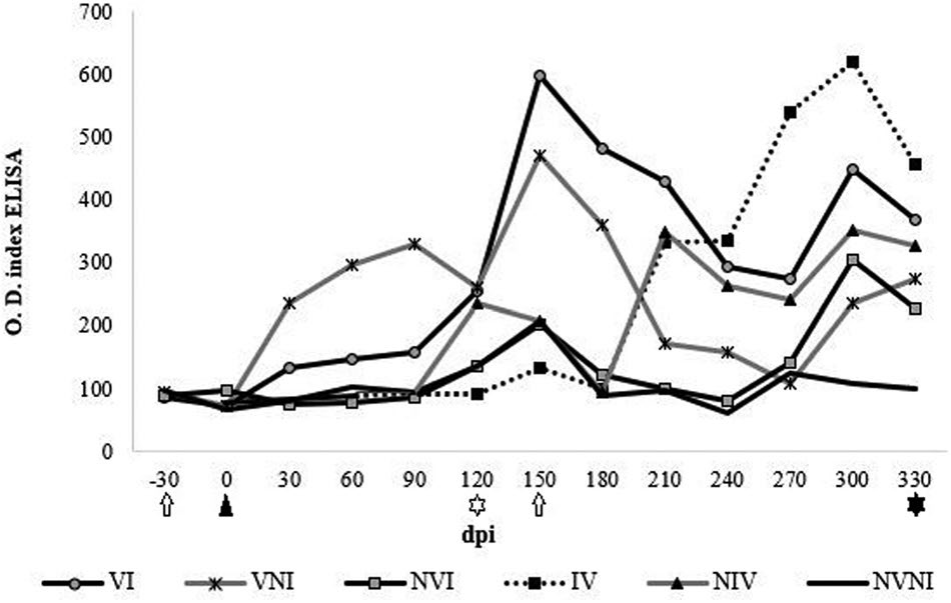
Evolution of the specific antibody against Map in the different experimental groups throughout the study. Values are expressed in different time-points as the mean of the OD quotients (OD of each sample/the OD of the positive control) within each experimental group (arithmetic mean ± SD). VI: vaccinated at −30 dpi and infected; VNI: vaccinated at – 30 dpi and not infected; NVI: not vaccinated and infected; NVNI: not vaccinated and not infected. IV: infected and vaccinated at 150 dpi; NIV; not infected and vaccinated at 150 dpi O.D.: Optical density quotient (*y* axis); dpi: days post-infection (*x* axis). White arrow: Vaccination (30 days prior to infection and 150 dpi; black arrowhead: infection (0 dpi); black rhombus: cervical intradermal skin test (90 dpi); white star: first slaughter (120 dpi); black star: second slaughter (330 dpi. Antibody production was significantly higher in animals in groups VI and VNI than in those in groups NVI and NVNI between 90 and 210 dpi (*p* < 0.05).

Groups vaccinated (VI and VNI) at −30 dpi (0 dpv) showed higher levels of antibodies against Map (*p* < 0.05) than the unvaccinated groups (NVI and NVNI) between 90 dpi (120 dpv) and 210 dpi (240 dpv) (Figure 4). In both vaccinated groups, the highest levels were found at 150 dpi (180 dpv). Although in the first months the ELISA indexes values were higher in the VNI group, they were surpassed by those of group VI at 150 dpi, although these differences were not statistically significant.

In the case of the groups vaccinated at 150 dpi (IV and NIV), the average ELISA indexes between 210 dpi (240 dpv) and 300 dpi (330 dpv) were significantly higher in IV than those of the unvaccinated (NVI and NVNI) and NIV. The highest levels were observed in NVI and IV groups at 300 dpi (Figure 4). A kid with macroscopic lesions presented the highest production of serum antibodies compared to the rest of the animals in the NVI group (individual data not shown). When comparing the groups vaccinated at −30 dpi (0 dpv) or 150 dpi (180 dpv), in both cases the differences in the ELISA index with their respective unvaccinated groups were maintained for 7 months after vaccination at −30 dpi and 3 months after vaccination at 150 dpi. Regarding the NVI group, only a significant increase in the mean values of the ELISA index was observed with the NVNI group from 300 dpi (*p* < 0.05), maintaining the increase in the last sampling (330 dpi), but not being significant (Figure 4).

The SICCT, which was carried out at 90 dpi (120 dpv) did not produce any significant change in the production of antibodies 1 month after its completion. However, at 2 months (150 dpi), there was an increase in the production of antibodies in groups VI, VNI, NVI and NVNI compared to the previous month (Figure 4).

#### 3.2.4. Characterization of lymphocyte subpopulations in peripheral blood

Although the data is not displayed individually, great variability was observed between animals of all groups. After the statistical analysis of the results, it could be seen that, in all the experimental groups and in the two samplings (at 120 and 330 dpi), the major subpopulation was γδT lymphocytes (immunostained with the WC1 antibody). Only significant differences were found (*p* < 0.05) between the VNI group with respect to the NVNI at 120 dpi (Supplementary Figure 1).

### 3.3. Pathological studies

**Figure 5.**
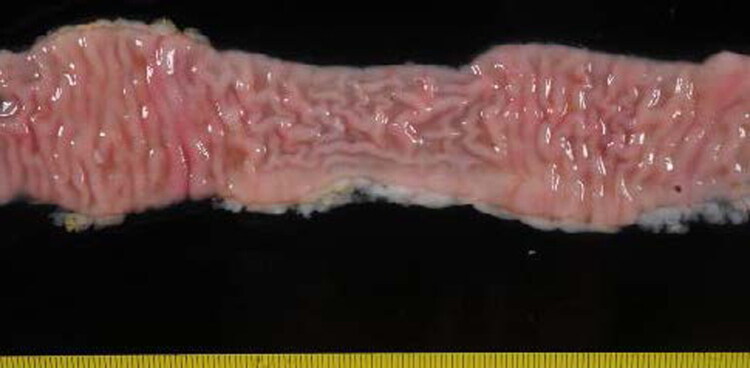
Section of the jejunum of the animal from group VI (330 dpi) showing diffuse thickening of the intestinal mucosa and highest level of antibodies against Map in serum.

The only animal with macroscopic lesions belonged to the infected and vaccinated group (IV) at 150 dpi and was slaughtered at 330 dpi. The main lesion observed was located in the jejunum, where a diffuse thickening of the mucosa was noted (Figure 5). The mesenteric lymph nodes were enlarged and exhibited diffuse thickening of the cortex.

#### 3.3.1. Histopathological study in animals slaughtered at 120 dpi

No lesions were found in any of the animals belonging to the non-infected groups. All the slaughtered kids (5/5) in the NVI (120) group and most of the slaughtered kids (2/3) from group VI (120) showed lesions compatible with paratuberculosis (Table 2).

**Table 2.** Distribution of the number of animals based on the type of lesion in both infected groups at 120 and 330 dpi.

	Slaughter at 120 dpi		Slaughter at 330 dpi		
Type of lesion	NVI	VI	NVI	VI	IV
No lesion	–	1/3	–	2/4	1/5
Focal	2/5	1/3	–	2/4	1/5
Multifocal a	2/5	1/3	1/3	–	–
Multifocal b	1/5	–	2/3	–	2/5
Diffuse	–	–	–	–	1/5

NVI: not vaccinated and infected; VI: vaccinated and infected; IV: infected and then vaccinated. Dpi: days post-infection.

**Figure 6.**
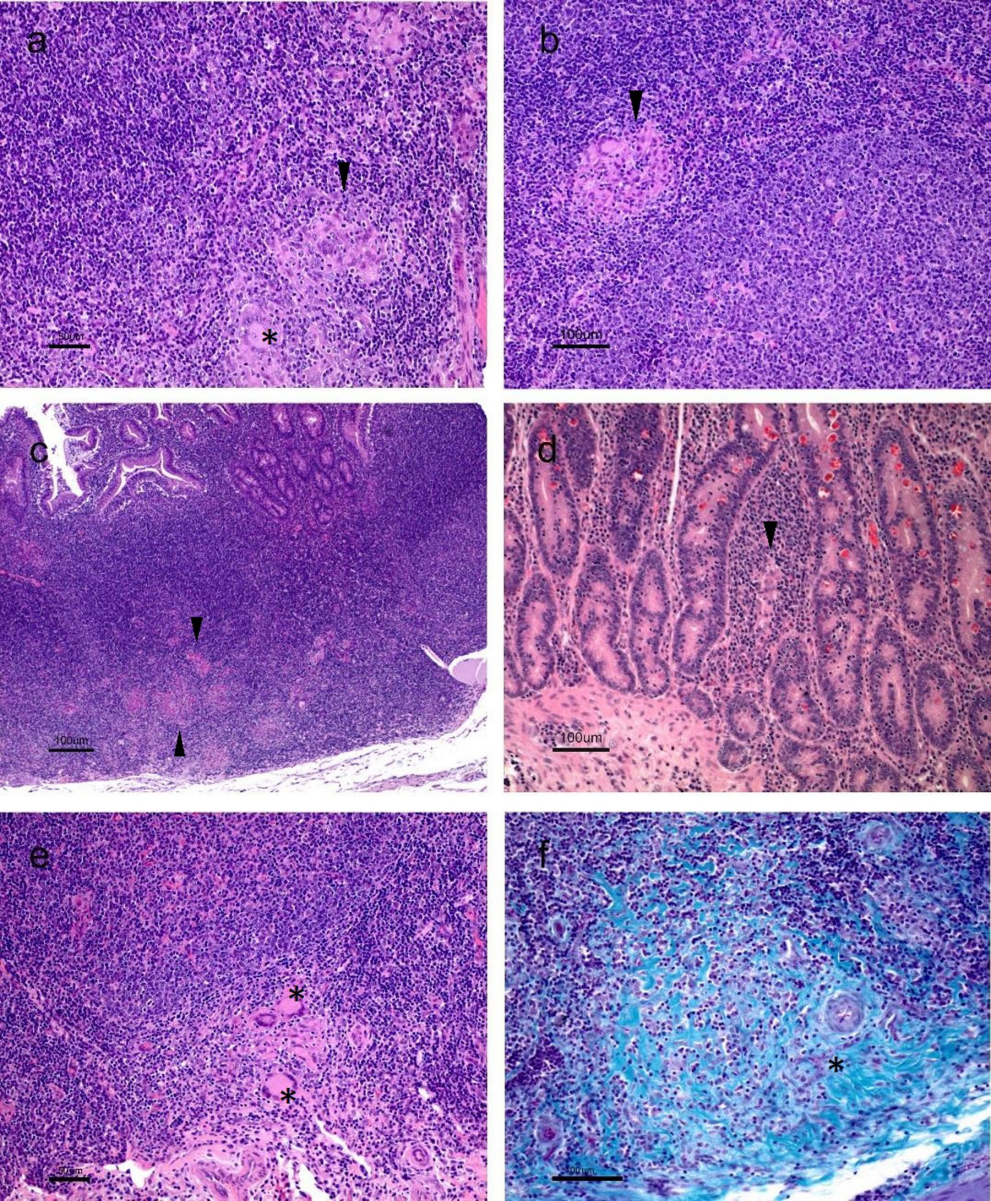
(a) Focal lesion of the NVI. 120 dpi. Poorly defined and infiltrative granuloma (arrowhead) with giant cell (asterisk). Jejunum. HE. (b) focal lesion in an animal from group VI. Well-defined granuloma composed by macrophages and giant cells (arrowhead). Jejunum. HE. (c) Multifocal lesion of the NVI group. Several granulomas located in the interfollicular area of jejunal Peyer’s patch, which begin to invade the lymphoid follicles. Granulomas are seen in the lamina propria adjacent to the lymphoid tissue (arrowhead). Jejunum. HE. d) Multifocal lesion b of the NVI group. Granuloma in lamina propria not adjacent to lymphoid tissue (arrowhead) Jejunum. HE. (e) and (f) Focal lesion found in an animal from group IV. Detail of a granuloma with HE (asterisks) and Masson’s trichrome stain where a large amount of connective tissue can be observed, infiltrating the jejunal granuloma (asterisk).

Particularly, kids from NVI (120) and VI (120) groups, focal lesions limited to the interfollicular area of the jejunum (Figure 6a and 6b) were observed in two and one animals, respectively, with the granulomas being always poorly delimited, showing an infiltrative character. Two other kids from NVI had a multifocal type a lesion (Figure 6c), with more abundant granulomas in the jejunal lamina propria, adjacent to the lymphoid tissue. The remaining animal showed a multifocal type b lesion (Figure 6d), which, together with severe Peyer’s patches lesion, also showed focal granulomas of scarce entity in areas of the proximal jejunum and proximal ileum not related to lymphoid tissue. Furthermore, in the kids in this group with multifocal lesion (type a or b), well-defined, multifocal distribution granulomas were also found in the lymph nodes (granulomatous lymphadenitis), located mainly in the paracortical region. Although they sometimes merged, they did not alter the normal structure of the organ.

However, in group VI (120), kids showed no lesions, either focal type (Figure 6b) with well-defined granulomas formed by macrophages and giant cells (Figure 6e), or multifocal type a lesion. It should be noted that most of the granulomas from jejunal lymph node showed fibrosis, with abundant collagen fibers observed between the cells that formed the granuloma (Figure 6f).

#### 3.3.2. Histopathological study in animals slaughtered at 330 dpi

As in the previous slaughter, lesions associated with Map infection were found in all the infected groups. In group VI, only 2 of the 4 animals examined (50%) presented lesions, while in the NVI and IV groups, they appeared in 100% and 80%, respectively (Table 2).

In the NVI group, a kid presented a multifocal a lesion, with abundant granulomas in the jejunum, generally larger than those found in the first sacrifice, adjacent to the lamina propria, invading the lymphoid follicles (Supplementary Figure 2a). The remaining two showed multifocal type b lesion, with a greater number of granulomas in jejunal areas without Peyer’s patches, not adjacent to lymphoid tissue (Supplementary Figure 2b and 2c), more in the animal of the first sacrifice. In those animals, moderate granulomatous lymphadenitis, with numerous granulomas in the paracortical and cortical areas of the jejunal lymph nodes, were also observed (Supplementary Figure 2d). Furthermore, one of them also showed serositis, with granulomatous lymphangitis in the outermost layer of the jejunal serosa (Supplementary Figure 2e). AFB were not observed by ZN staining. In the two animals from VI group, a focal lesion in the jejunum, with abundant connective tissue as seen in the previous group, was also observed (Supplementary Figure 2f).

Finally, animals from group IV showed great variability related to the type of lesion (Table 2). In one of them, the focal lesion, with small, well-defined granulomas, also fibrosed, appeared in the jejunal lymphoid tissue (Supplementary Figure 3a). Two other animals presented multifocal type b lesions, in which the granulomas had already spread to areas of the jejunal lamina propria, whether adjacent or not to the lymphoid tissue; although these were limited of extent, they did not significantly alter the structure of the intestinal wall (Supplementary Figure 3b and 3c). In these two kids, granulomatous lymphadenitis could also be observed in the lymph nodes associated with areas of necrosis (Supplementary Figure 3d).

**Figure 7.**
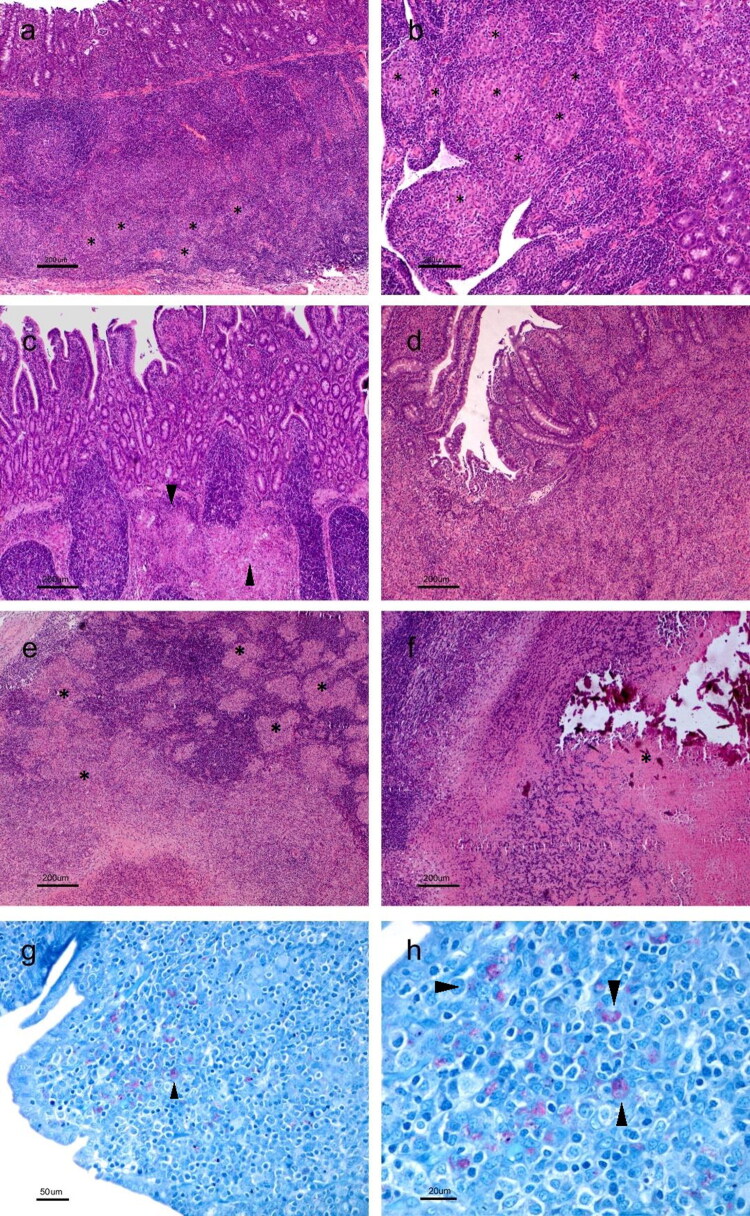
Lesions found in IV group. (a) Granulomas altering the normal structure of the submucosa of the ileum (asterisks). HE. (b) Detail of a thickening of the ileum microvilli with number of granulomas (asterisks). HE. (c) Granuloma altering the normal structure of the submucosa of the ileum and dilatation of the lymphatic vessels (arrowhead). HE. (d) Granulomas alter the structure of both the mucosa and submucosa of the ileocecal valve, causing a thickening of the microvilli. HE. (e) Multiple granulomas in the cortical and medullary of a jejunal lymph node (asterisks). (f) granulomas converge on the right side of the image, forming a large granuloma that presents caseous necrosis and areas of mineralization in its center (asterisk). HE. (g) and (h) Presence of AFB in the cytoplasm of macrophages associated with a diffuse jejunal injury (arrowheads). ZN.

The remaining animal showed a diffuse multibacillary lesion characterized by a diffuse macrophage infiltrate in some jejunal Peyer’s patches, completely altering its structure (Figure 7a and 7b). The same type of cells was found in areas of the lamina propria of various areas of the jejunum and ileum without lymphoid tissue, causing thickening of the villi (Figure 7c and 7d). This animal was the only one with a macroscopically visible multifocal thickening of the jejunal mucosa (Figure 5). Additionally, diffuse granulomatous lesions were observed in this animal throughout the section of the regional jejunal lymph nodes, where the granulomas converged and altered the normal histological structure (Figure 7e), both cortical and medullary, with some areas displaying necrosis (Figure 7f). The presence of AFB inside the macrophages was detected by ZN staining (Figure 7g and 7h). This animal is the same one that showed an increase in the release of IFN-γ against avian PPD and in the production of serum antibodies in the months prior to slaughter.

#### 3.3.3. Granuloma counts in animals slaughtered at 120 and 330 dpi

**Figure 8.**
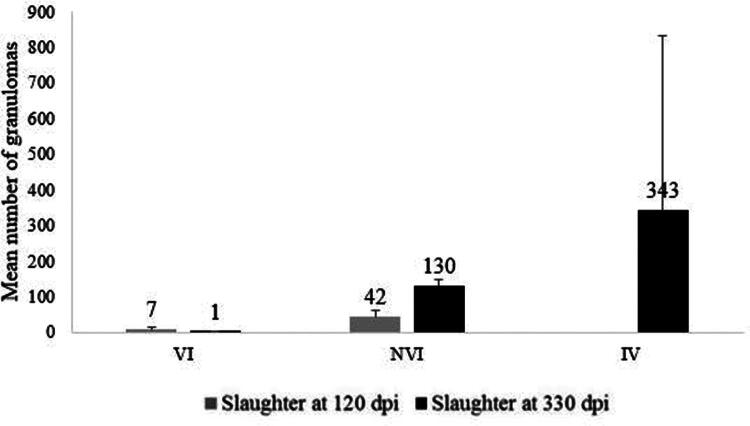
Mean number of granulomas (arithmetic mean ± SD) found in the different experimental groups (VI, IV and NVI) slaughtered at 120 dpi and 330 dpi. VI: vaccinated at −30 dpi and infected at 0 dpi; NVI: Not vaccinated and infected at 0 dpi; IV: infected at 0 dpi and vaccinated at 150 dpi.

In order to characterize the intensity of the injuries associated with Map and to be able to establish its statistical comparison between the different groups and the slaughter time-point, a count of the number of granulomas in the intestine and jejunal lymph nodes was performed. The results of the total number of granulomas per animal expressed as the average value for each experimental group, are shown in Figure 8. In the slaughter at 120 dpi, the mean number of granulomas of each animal in the NVI group (42) was significantly higher (*p* < 0.05) than the mean number of granulomas in the group VI kids (7). A similar result, although in this case with higher significant differences (*p* < 0.0001), was observed in the second sacrifice (330 dpi) when comparing both groups (130 granulomas in the NVI group vs. 1 in the VI group). At 330 dpi during the second slaughter, the average number of granulomas in animals from the IV did not differ significantly from those in NVI group. However, there was a statistically significant difference in the number of granulomas when comparing the IV to the VI group (Figure 8).

When the evolution of the number of granulomas was analyzed, between both slaughter moments, within the animals of each experimental group, the granuloma counts in the NVI group increased considerably (*p* < 0.05) while total count of lesions from VI group decreased at 330 dpi (Figure 8).

**Figure 9.**
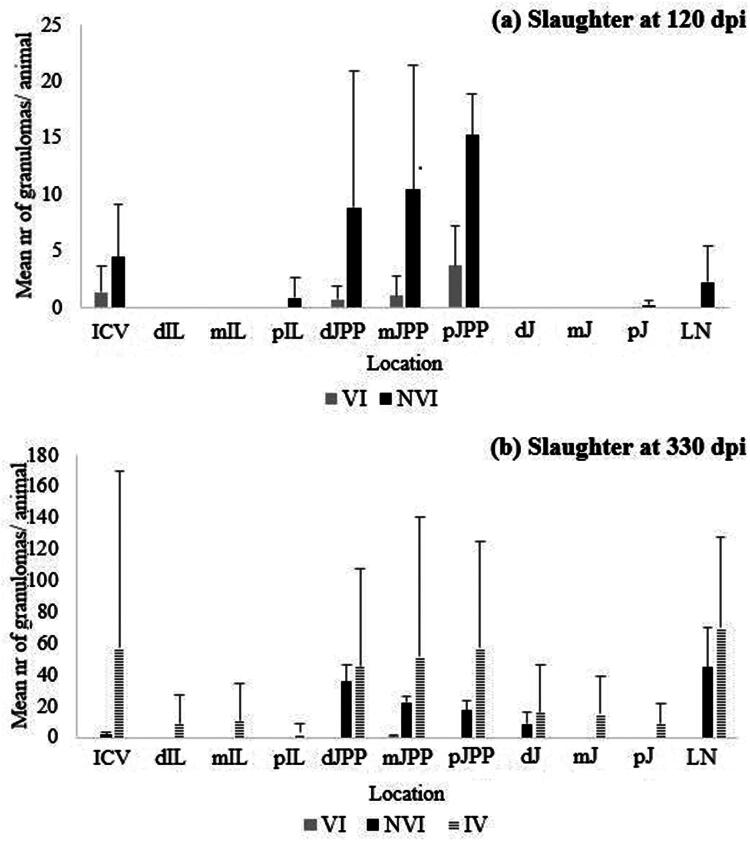
Mean number of granulomas count per group found in (a) the first slaughter at 120 dpi and (b) second slaughter at 330 dpi for each of the locations from examined small intestine and lymph nodes according to experimental groups (arithmetic mean ± SD). VI: vaccinated at −30 dpi and infected; NVI: not vaccinated; IV: infected at 0 dpi and vaccinated at 150 dpi; ICV: ileocecal valve; dIL: distal ileum; mIL: mid ileum; pIL: proximal ileum; dJPP: distal jejunal Peyer’s patch; mJPP: mid jejunal Peyer’s patch; pJPP: proximal jejunal Peyer’s patch; dJ: distal jejunum; mJ: medium jejunum; pJ: proximal jejunum; LN: lymph nodes. Mean number of granulomas index (*y* axis). Significantly different (*p* < 0.05) between VNI and NVNI groups at 120 dpi groups are identified with an asterisk (*).

According to their location in the different sections of intestine and lymph nodes examined, the granulomas observed in both the animals of the NVI and VI groups were located mainly in the lymphoid and jejunal tissue (JPP) but were always more abundant (*p* < 0.05) in the NVI group, without differences between the jejunal areas. Subsequently, they were also found, but in smaller numbers than in JPP (*p* < 0.05), in the ileocecal valve, where they were also significantly more numerous (*p* < 0.05) in the NVI group. In the jejunal lymph node, only lesions were found in the NVI group (Figure 9a).

The kids in the NVI group at the second sacrifice, at 330 dpi, showed granulomatous lesions in all the sampled lymphoid sites (ICV, IL JPP, LN), being significantly more numerous in JPP and LN than in the ICV (*p* < 0.05) (Figure 9b). In addition, granulomas were also found in samples of the distal jejunum without Peyer’s patches (dJ) in less number than in the JPP. However, in group VI, granulomas, significantly less than the animals in the NVI group (*p* < 0.05), were only found in the lymphoid tissue of the middle jejunum (mJPP). In the case of group IV, granulomas were found in all the tissues sampled, although no significant differences were detected with the NVI group (Figure 9b). No granulomas were observed in the duodenum, neither in the cecum nor in the rectum.

### 3.4. Microbiological studies

#### 3.4.1. Bacterial culture

Positive isolates were only obtained from feces in one animal from group NVI, which presented multifocal b lesions and in tissue from an animal from group IV with diffuse lesion, slaughtered at 330 dpi only in tubes seeded with the HEYM medium (Supplementary Table 1).

#### 3.4.2. Nested PCR

Map DNA was not detected in any animal from the uninfected groups (VNI, NIV, and NVNI) throughout the study. In the first slaughter (120 dpi), mycobacterial DNA from jejunum samples of goats in the NVI group with multifocal a and b lesion were identified, while in group VI the only positive samples were ICV and LN that belonged to a kid with multifocal and focal lesion. In the slaughter moment (330 dpi), Map DNA could be identified in 100% of the goats in the NVI and VI groups (including the two animals without lesions). In both slaughter points the locations with the highest frequency of positive results were the middle jejunum with Peyer’s patches and the lymph nodes. In the case of group IV, it was possible to identify Map DNA in 80% of the goats in at least two samples analyzed. The only animal in this group where Map could not be detected was the one without histological lesions (Supplementary Table 1).

### 3.5. Local immune response determination

The study of the local immune response was carried out by quantifying the specific production of IFN-γ and characterizing the lymphocyte subpopulations present in areas of intestinal lymphoid tissue and lymph nodes, after extraction of mononuclear leukocytes from them.

#### 3.5.1. *In vitro* IFN-γ secretion

**Figure 10.**
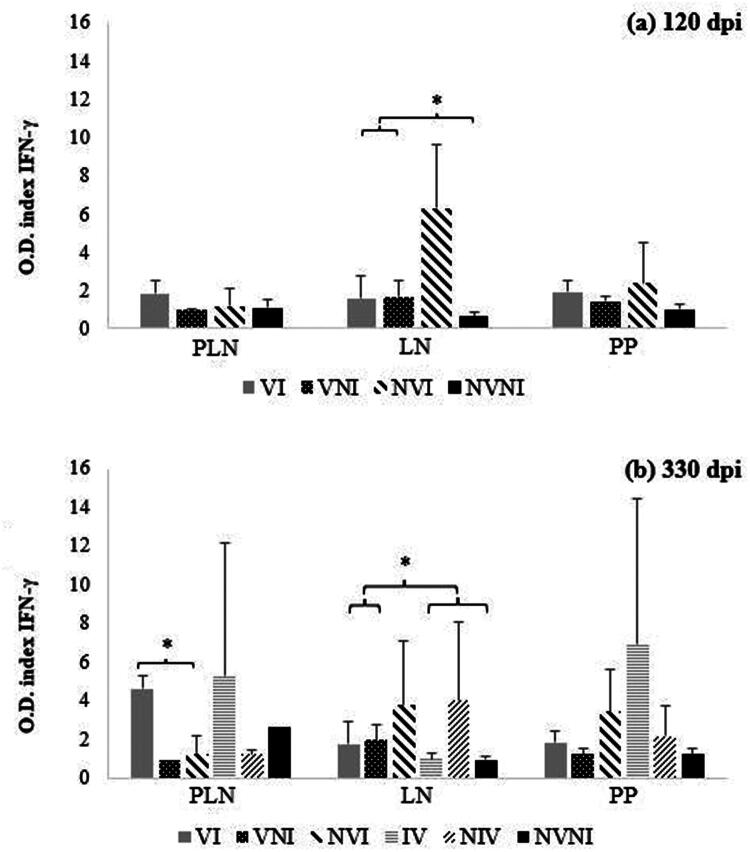
Results of mean values of *in vitro* IFN-γ secretion in different tissue samples at (a) 120 and (b) 330 dpi from different experimental groups, after stimulation with avian PPD (arithmetic mean ± SD). PLN: prescapular lymph node; LN: lymph nodes associated to the gut; PP: Peyer’s patches; VI: vaccinated and infected; VNI: vaccinated and not infected; NVI: not vaccinated and infected; IV: infected and vaccinated at 150 dpi; NIV: non-infected and vaccinated at 150 dpi; NVNI: not vaccinated nor infected. IFN-γ index (*y* axis). Significantly different (*p* < 0.05) between groups are identified with an asterisk (*).

The determination of the *in vitro* release of IFN-γ was carried out in five tissue samples (prescapular lymph node (PLN), caudal jejunal lymph node (CJLN), medium jejunal lymph node (MJLN), jejunal Peyer’s patch (JPP) and ileum). Although the five samples were processed individually, to simplify the analysis of results, those results obtained in the Peyer’s patches of the jejunum and ileum were grouped into a single data as PP, as well as the values obtained from the caudal jejunal and medial jejunal lymph nodes, which were analyzed together as LN (Figure 10a and 10b).

The values of the IFN-γ index after the stimulation of the mononuclear leukocytes isolated from the PLN with avian PPD, did not show significant differences between the animals of the different groups sacrificed at 120 dpi (150 dpv). However, in those sacrificed at 330 dpi (360 dpv), the values were higher (*p* < 0.05) in the samples of group VI compared to those of the NVI group (Figure 10b).

Concerning the intestinal and lymph node samples, the NVI group showed significant differences (*p* < 0.05) with higher values compared to the other groups in the LN samples, both at the first and second slaughter, except for the NIV group during the second slaughter. However, no differences were observed in the results from the PP samples for the animals examined at either slaughter, likely due to the considerable variability in lesions (Figure 10a and 10b).

#### 3.5.2. Characterization of lymphocyte subpopulations in tissues

In the study, results from jejunum Peyer’s patches and ileum were grouped as PP, while those from the caudal jejunum and mid jejunum lymph nodes were grouped as LN. CD21 (B-lymphocytes) were the predominant lymphocytes in all samples analyzed. In the PLN samples, no significant differences were observed between groups or time-points. CD4+ and CD8+ lymphocyte populations increased between the first and second slaughters in both PP and LN, irrespective of treatment. Neither vaccination nor challenge appeared to significantly affect the percentages of lymphocyte subpopulations.

## 4. Discussion

The present study evaluated the effect of vaccination against paratuberculosis in an experimental goat model with the objective of analyzing both possible effects: protective (immunization before infection) and therapeutic (vaccination of infected animals), assessing pathogenesis, including local and peripheral immune responses. The experimental model allows us to assess these effects in the initial phases of Map infection and has demonstrated that this study time-point (330 dpi) is enough to determine the influence of different factors, such as vaccination, on paratuberculosis pathogenesis (Delgado et al. 2013; Fernández et al. 2014; Muñoz 2014, Windsor et al. 2014; Stefanova et al. 2024). Moreover, the caprine species has been chosen because its response to mycobacterial infections presents similarities with that of the bovine species and both species are susceptible to tuberculosis infection. In most of the studies, Gudair^®^ vaccine, registered for caprine use, has been employed. However, in this study, Silirum^®^ vaccine, registered for bovines, has been employed. This vaccine has been shown to have a protective effect in caprine species (Hines et al. 2014) and the findings from this study may offer insights that could be studied in cattle.

Vaccination is regarded as the most effective strategy for controlling paratuberculosis, considering its favorable cost-benefit ratio and its ability to provide protection against infection (Bastida and Juste 2011; Groenendaal et al. 2015). It reduces the number of clinical cases and decreases the *Mycobacterium avium* subsp. *paratuberculosis* (Map) excretion, although the immune response varies among animals. Our study corroborates that vaccination provides protection, mainly limiting to focal lesions at most compared to those animals that were infected but not vaccinated, with multifocal forms not related to lymphoid tissues. Despite this protective effect, substantial individual variability was observed, with some goats showing no lesions while others exhibited multifocal lesions, similar to observations under field conditions (Pérez et al. 1995; Corpa et al. 2000b, 2000c; Reddacliff et al. 2006; de Silva et al. 2015; Windsor, 2015). No clinical signs were detected in any case, which is expected, the second slaughter occurred at 330 days post-infection (dpi), and paratuberculosis generally has a longer subclinical course prior to the manifestation of clinical signs (Chiodini et al. 1984; Clarke, 1997; Dennis et al. 2011).

In the present study, in line with other experimental studies in bovine (Muñoz, 2014), ovine (Juste et al. 1994) or caprine species (Storset et al. 2001), vaccination 1 month before infection has demonstrated that it does not prevent Map infection, but it does produce a clear benefit by controlling the development of the lesion into more severe forms, which are commonly observed in advanced stages of the disease (Chiodini et al. 1984; Clarke 1997; Dennis et al. 2011), as observed in the VI group compared with the NVI. Most lesions belong to the focal forms and were restricted to lymphoid tissue and could be associated with paratuberculosis protection or latency forms (Pérez et al. 1996; Sigurdardóttir et al. 1999; Corpa et al. 2000a; Storset et al. 2001; de Silva et al. 2015; Arrazuria et al. 2016). The morphology of granulomas was also different between both groups; in the VI group, granulomas were well-demarcated with fibrosis while in the NVI group, granulomas had an infiltrative character. Well-defined granulomas surrounded by connective tissue have been associated to protection forms of paratuberculosis (Juste et al. 1994; Delgado et al. 2013; Fernández et al. 2014) and tuberculosis (García-Jiménez et al. 2012) and, also with regressive forms, with involution of the granulomas (Juste et al. 1994; Delgado et al. 2013; Fernández et al. 2014). The results of this study indicate that vaccination causes the containment of infection in focal forms, limiting the progression to more severe forms and, later on, decreases the number of lesions, since these were significantly lower in the second slaughter compared to the first. This effect has already been suggested in previous studies, both in experimental and field conditions, in the caprine species (Corpa et al. 2000b; Hines et al. 2014), bovine species (Sweeney et al. 2009; Muñoz, 2014), and ovine species (Juste et al. 1994; Pérez et al. 1995).

Another interesting point of this work was to analyze the effect of vaccination on the specific immune response against Map. Vaccination at 30 days before infection (−30 dpi) triggered an intense cellular and humoral response. This effect was described before in goats (Corpa et al. 2000b; Chartier et al. 2012), rabbits (Arrazuria et al. 2016), bovines (Muñoz, 2014; Tewari et al. 2014) and sheep (Juste et al. 1994; Corpa et al. 2000b; Begg and Griffin 2005). The cellular immune response was evaluated at 120 dpv (90 dpi) by the SICCT. According to this test, all animals were negative for tuberculosis, demonstrating the value of this test to differentiate paratuberculosis vaccinated animals from tuberculosis-infected animals, at least in tuberculosis-free farms (Chartier et al. 2012; Coad et al. 2013; Garrido, 2013). However, if the single intradermal test (SICT)) had been used alone, interference with tuberculosis diagnosis would have occurred because seven vaccinated goats were positive with this technique. In a field study, also in goats (Chartier et al. 2012) at 8 months post-vaccination, these interferences were reduced, although they remained detectable in some animals. These results indicate that the SICCT can be employed in the goat species to eradicate tuberculosis, at least during the first months after vaccination (Coad et al. 2013; Garrido et al. 2013; Muñoz, 2014). Johnin was also assessed, together with avian PPD, but contrary to previous studies (Köhler et al. 2001; Nedrow et al. 2007), in our case it did not offer better results.

Besides SICCT, cellular immune response based on IFN-γ released was assessed by IGRA. This cytokine is one of the most important in the cellular response against Map (Stabel, 2006; Begg et al. 2011) and high levels of antigen-specific interferon-producing cells in peripheral blood have been associated to protection against Map infection (de Silva et al. 2010). Vaccination at −30 dpi increased IFN-γ levels, as previous studies had demonstrated (Gwozdz et al. 2000a; Muñoz, 2014; Ghosh et al. 2015) approximately between 1 month and 2 months post vaccination (Gwozdz et al. 2000b; Corpa et al. 2000b; García-Pariente et al. 2003; Muñoz, 2014; Arrazuria et al. 2016). This increase was always higher when blood samples were stimulated with avian PPD than with bovine PPD, in agreement with previous studies (Muskens et al. 2002; Pérez de Val et al. 2012; Arrazuria et al. 2016).

The cellular immune response, particularly the elevated levels of IFN-γ, has been linked to protective mechanisms in paratuberculosis (Stabel, 2000; Begg and Griffin 2005; Koets et al. 2015). Overall, the results obtained corroborate the hypothesis, as the VI group exhibited significantly increased levels of IFN-γ associated with these protective responses. The vaccinated groups – VNI, VI, IV, and NIV – demonstrated a robust immune response (IFN-γ) 1-month post-vaccination. Conversely, animals that were infected (either prior to or following vaccination) displayed lower levels of IFN-γ than the non-infected groups. This suggests that the vaccine alone induces a strong, specific immune response that is more easily quantified when not impacted by the effects of active infection. The high infection dose present in the infected groups may compromise the immune response, diverting it toward regulatory pathways or limiting the activation of effector T cells stimulated by the vaccine. Consequently, the presence of high-dose infection, whether occurring before or after vaccination, may impede the optimal activation and proliferation of immune cells in a short time. This leads to a delayed immune response compared to that observed in vaccinated animals that have not experienced infection. However, when results are analyzed in more detail in the NVI group, some animals have presented focal forms at 120 dpi, associated to initial phases (Juste et al. 1994; Fernández et al. 2014) or protection-latency of Map infection (Pérez et al. 1996; Corpa et al. 2000a; Vázquez et al. 2013), without increased IFN-γ levels. This finding has been previously documented in both experimental and field conditions by several authors (Begg et al. 2011; Vázquez et al. 2013; Fernández et al. 2017). An elevated peripheral cellular immune response may indicate previous exposure to Map antigens through either infection or vaccination. However, it is important to note that this response does not necessarily equate to protection against Map infection. Additionally, our findings suggest a correlation between the intensity of the immune response and the development of lesions. Within the NVI group, there is a continuous increase in antibody levels throughout the infection, particularly among animals with multifocal a and multifocal b forms.

This study has also evaluated the influence of vaccination on humoral immune response by indirect ELISA. An increase in antibody levels was observed at 30 dpi (60 dpv) and 60 dpi (90 dpv) in animals vaccinated at −30 dpi and in IV and NIV groups respectively and maintained until 7 months. There are several studies where paratuberculosis vaccination has proved to induce an intense humoral immune response (Muskens et al. 2002; García-Pariente et al. 2003; Begg and Griffin 2005; Hines et al. 2007; Coad et al. 2013; Hines et al. 2014; Tewari et al. 2014). In some studies, carried out in the caprine species with Gudair^®^, the duration of the response was like that of this work (Corpa et al. 2000b) or even longer (Singh et al. 2007). In a study conducted in goats using the same vaccine (Hines et al. 2014), the vaccination effects were observed to last for 13 months. It is important to note that serological diagnosis for paratuberculosis is not reliably accurate for at least 3 months following vaccination. High antibody levels are typically associated with advanced lesions and the presence of acid-fast bacilli (AFB) (Clarke 1997; Pérez et al. 1997; Storset et al. 2001; Vázquez et al. 2013). Recent research has suggested that antibodies may also contribute to the immune response against Map (Jolly et al. 2022). This may explain the elevated antibody levels observed in the vaccinated group (VI). However, in the non-vaccinated infected group (NVI), high antibody levels were detected in animals with advanced lesions that is consistent with observations from natural of infection (Pérez et al. 1997; Vázquez et al. 2013). Thus, the presence of antibodies appears to correlate with the progression of Map infection in infected, non-vaccinated animals, resulting in the development of more extensive lesions as antibody levels began to rise toward the end of the study. Conversely, high levels of antibody production observed in vaccinated animals in the absence of lesions serve as an indicator of effective vaccination.

Additionally, the effect of vaccination on subpopulations of lymphocytes in peripheral blood was evaluated by flow cytometry. In previous studies (Mateo et al. 1997; Corpa et al. 2000a; Hasvold et al. 2002; Gillan et al. 2010; de Silva et al. 2015) variations in the levels of lymphocytes subpopulations associated to vaccination have been found, but results have been contradictory. For example, while in some of them, levels of CD4+ T lymphocytes were increased (de Silva et al. 2015), in others, these were decreased (Gillan et al. 2010). In this study, the most remarkable result was the high individual variability observed. As a result, significant differences in lymphocyte subpopulations were not detected, and therefore, the effect of vaccination could not be confirmed. A study including a higher number of animals would be necessary to be conclusive. γδ T lymphocytes constituted the predominant subpopulation of lymphocytes in the peripheral blood, participating in the initial phases of the innate immune response to intracellular infections (Pollock and Welsh 2002), particularly in the organization of granulomas (Tanaka et al. 2000), which accounts for their elevated presence in animals exhibiting focal or multifocal lesions. Furthermore, γδ T cells constitute the primary lymphocyte population in the peripheral blood of calves (Hein and Mackay, 1991) and in goats, as indicated by the findings of this study. They are also abundant in epithelial tissues, serving as the first line of defense against microbial invasion (Hayday, 2000). However, the specific contributions of these cells in this context and their roles require further investigation to be fully validated. In addition to the peripheral immune response, variations in the local immune response were assessed through the analysis of lymphocyte subpopulation levels in intestinal lymphoid tissue and lymph nodes using flow cytometry. In Peyer’s Patches and lymph nodes of ruminants, B-lymphocytes are the predominant population (Reynolds et al. 1991), while T lymphocytes are present in the interfollicular areas (Reynolds et al. 1991) are less numerous. Conversely, γδ T lymphocytes are less abundant in lymphoid tissue compared to peripheral blood. The proportions of CD4+ and CD8+ lymphocytes do not differ significantly across all locations between the NVNI group and the other groups of goats, therefore, there is no indication of an ongoing specific immune response of an acquired nature. Vaccination did not modify the IFN-γ release of mononuclear cells from Peyer’s patches and lymph nodes. Significant differences were only detected when mononuclear cells from the prescapular lymph node were stimulated with avian PPD in the VI group at 330 dpi (360 dpv). Higher levels were found in the second slaughtered (330 dpi), similar than other studies (Bannantine et al. 2022; Stefanova et al. 2024), when IFN-γ levels from peripheral blood started to decrease. This finding is not in agreement with a previous study developed in bovine (Sweeney et al. 2009) where the highest levels of IFN-γ secreted by lymph node and peripheral blood cells were found at 60 dpi. Similar to other studies in calves (Muñoz, 2014) and goats (Bannantine et al. 2022), the highest IFN-γ levels were found after stimulation of lymphocytes from the prescapular lymph node at 330 dpv. In any case, lymphocytes from the prescapular lymph node would collaborate in the IFN-γ release after vaccination. Vaccination does not seem to have a clear effect over lymphocytes from the intestinal lymphoid tissue, which did not participate in local production of IFN-γ where lesions were observed, contrarily to what was previously observed in calves (Muñoz, 2014), where vaccinated animals had higher levels than non-vaccinated animals. Although it might be a species difference, possibly the scant number of studied animals and the great individual variability can explain these different results. In the NVI group, a higher production of IFN-γ has been found in lymphocytes obtained from lymph nodes, in both slaughters, and in lymphoid tissue from Peyer’s patches in the second slaughter; so, infection would cause an immunomodulatory effect on lymphocytes from these locations and play a role in the interaction between Map and the host. Similar findings were found in calves at 300 dpi (Muñoz, 2014).

On the other hand, the effect of vaccination on fecal shedding and tissue colonization has been evaluated. Regarding Map shedding, the effect of vaccination could be positive as bacteria have only been isolated from feces in one animal of the NVI group with multifocal b lesions and in tissue from one of the IV group with diffuse lesions. However, the frequency of fecal samplings throughout the study was too low a proper assessment of fecal shedding given the fact that shedding may occur intermittently. In the case of tissues from the NVI groups animals, Map was not isolated in any of them but differences between the groups could only be assessed if the proportion of positive tissues by culture was significantly higher in the NVI animals than in all vaccinated and infected groups. However, it should be considered that it has only occurred in one animal, not in the rest.

Nested PCR showed greater sensitivity compared to bacteriological isolation as expected and previously documented (Collins et al. 1993; Gao et al. 2009; Soumya et al. 2009; Delgado et al. 2013; Muñoz, 2014). On the other hand, PCR detects fragments of nucleic acids of Map, so bacterial culture would be a technique to detect colonization by viable bacillus. All animals from the VI group were positive except for one slaughtered at 120 dpi. In the case of the NVI group, Map was also detected, although the number of samples was higher in the second slaughter (330 dpi). In infected and then vaccinated animals (IV), as previous results reflected, Map was identified in less locations than animals not vaccinated but infected (NVI), suggesting that vaccination may reduce Map colonization. In relation to histopathological examination, PCR would also be more sensitive because Map could be detected by PCR from infected animals without lesions. In any case, PCR from paraffin-embedded tissues would be a good complement to corroborate the specificity of granulomatous lesions, especially in focal lesions which are usually negative to other diagnosis techniques such as ZN or immunohistochemistry (Pérez et al. 1996; Delgado et al. 2013). However, it should be noted that the detection of Map in fecal samples could be influenced by the intermittent shedding of Map, especially in subclinical stages (Soumya et al. 2009; Stefanova et al. 2024).

Regarding infection, the VI and NVI groups exhibited significant individual variability in lesion presentation, which is consistent with previous experimental studies (Juste et al. 1994; Köhler et al. 2001; Storset et al. 2001; Delgado et al. 2013; Fernández et al. 2014). Notably, at the second slaughter, the NVI group displayed more extensive lesions, including diffuse lesions, compared to the first slaughter. This differs from observations in the VI group and other studies (Verna et al. 2007; Delgado et al. 2013; Krüger et al. 2015), where severe lesions were noted at 330 dpi or even earlier. Cross-study comparisons are challenging due to variations in animal species, breeds, inocula, and doses used. Some studies employed inoculum from the intestinal mucosa of clinically infected animals (Verna et al. 2007; Delgado et al. 2013), which tend to be more pathogenic than strains used under experimental conditions (Begg et al. 2010; Fernández et al. 2015). The K-10 strain used in this study, previously employed successfully (Stabel et al. 2009; Fernández et al. 2014), has lower pathogenicity compared to strains from clinical cases (Fernández et al. 2014). Lesions were predominantly found in the Peyer’s patches of the jejunum and the ileocecal valve, as documented in other studies (Sigurdardóttir et al. 1999; Storset et al. 2001; Verna et al. 2007; Delgado et al. 2013; Fernández et al. 2014; Muñoz, 2014; Stefanova et al. 2024). Furthermore, the observed immune responses and the progression of lesions from focal and multifocal to diffuse by the second slaughter underscore the reliability of this model. The mechanisms that induce these changes in granulomatous lesions depending on vaccination were not investigated in this work.

In conclusion, vaccination has important effects on the pathogenesis of paratuberculosis, influencing the type of lesions and their evolution, tending to reduction. However, the vaccination of previously infected animals has shown great variability in the response of the animals, including individuals with focal lesions or even no lesions coexisting with other animals with severe and abundant forms of AFB. Consequently, the study has not fulfilled the requirements to be able to reliably demonstrate an intended therapeutic effect in all cases; even these results suggest that it could contribute to entangling an infection already established in some animals. Vaccination has also shown its effect in terms of the protection against disease, especially in goats vaccinated before Map infection, limiting the achieving of severe lesions and identification of bacteria in feces and tissues, production of IFN-γ and antibodies in peripheral blood. Otherwise, it does not appear to affect the levels and type of lymphocyte populations circulating or present in the tissues.

## Supplementary Material

Supplementary_Material.docx
