# Supplementary material for: Effect of paratuberculosis vaccination before and after oral experimental infection with Mycobacterium avium subspecies paratuberculosis in goats
Source: Vet Q. 2025 Oct 1;45(1):2566363. doi: 10.1080/01652176.2025.2566363 (PMC12490372; doi:10.1080/01652176.2025.2566363)
Supplement: Supplementary_Material.docx [file TVEQ_A_2566363_SM4700.docx]

Supplementary Material

Effect of paratuberculosis vaccination before and after oral experimental infection with Mycobacterium avium subspecies paratuberculosis in goats

Marcos Royo^1,2^, Natalia Elguezábal^3^, Rakel Arrazuria^3^, Julio Benavides^2^, Miguel Fernández^1*^

^1^Departamento de Sanidad Animal, Facultad de Veterinaria, Campus de Vegazana, Universidad de León, 24007 León, Spain.

^2^Departamento de Sanidad Animal, Instituto de Ganadería de Montaña (CSIC-Universidad de León), 24346 Grulleros, Spain.

^3^Animal Health Department, NEIKER-Instituto Vasco de Investigación y Desarrollo Agrario, Basque Research and Technology Alliance (BRTA). Derio, Bizkaia, Basque Country, Spain.

*** Correspondence:**Miguel Fernández
m.fernandez@unileon.es


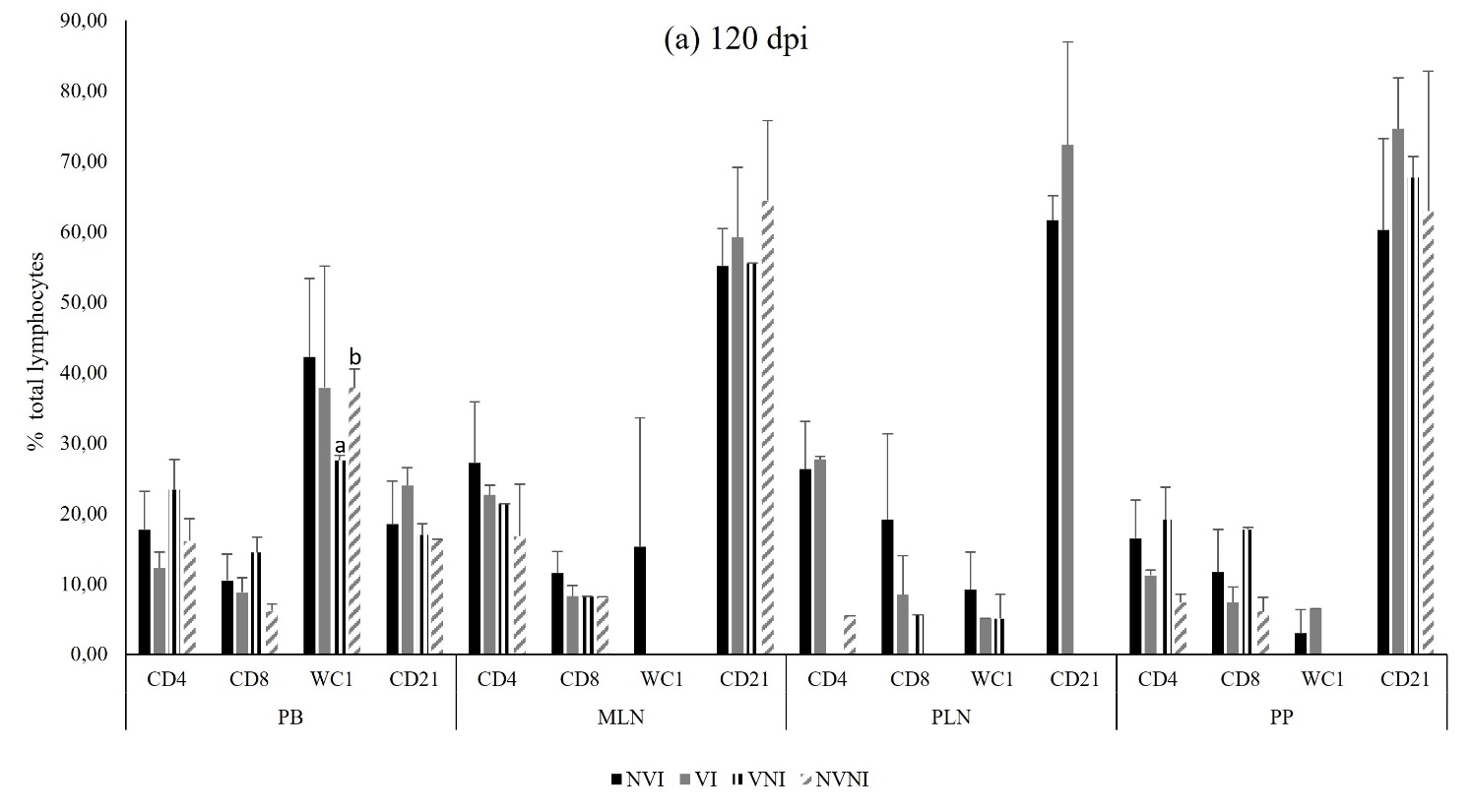


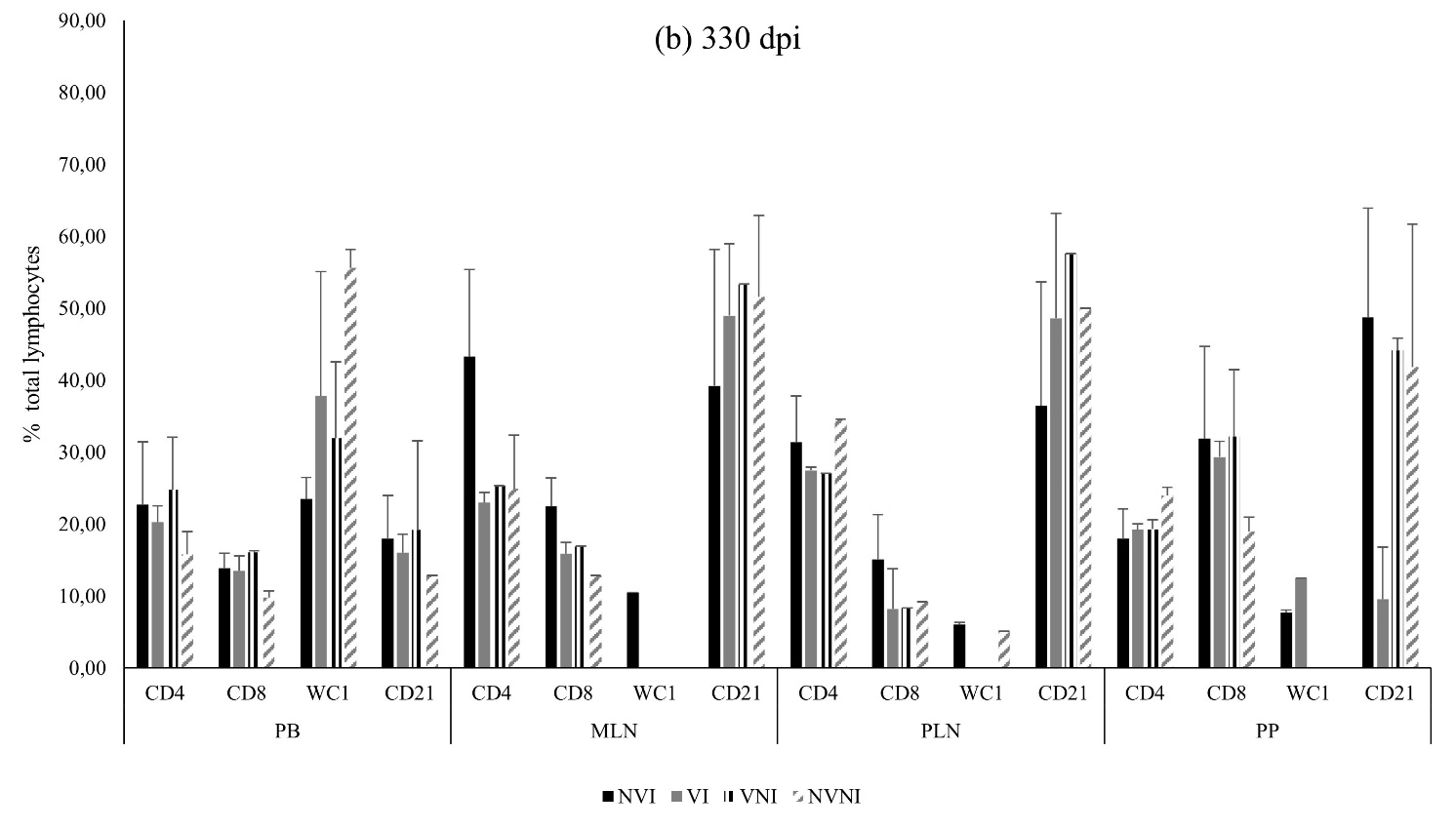


**Supplementary Figure 1**. Results of % of total lymphocytes population in peripheral blood (PB), mesenteric lymph node (MLN), preescapular lymph node (PLN) and Peyer’s patches (PP) at (a) 120 and (b) 330 dpi from different experimental groups. NVI: not vaccinated and infected; VI: vaccinated and infected; VNI: vaccinated and not infected; NVNI: not vaccinated nor infected. Significantly different (p<0.05) between groups are identified with *a* and *b* letters.


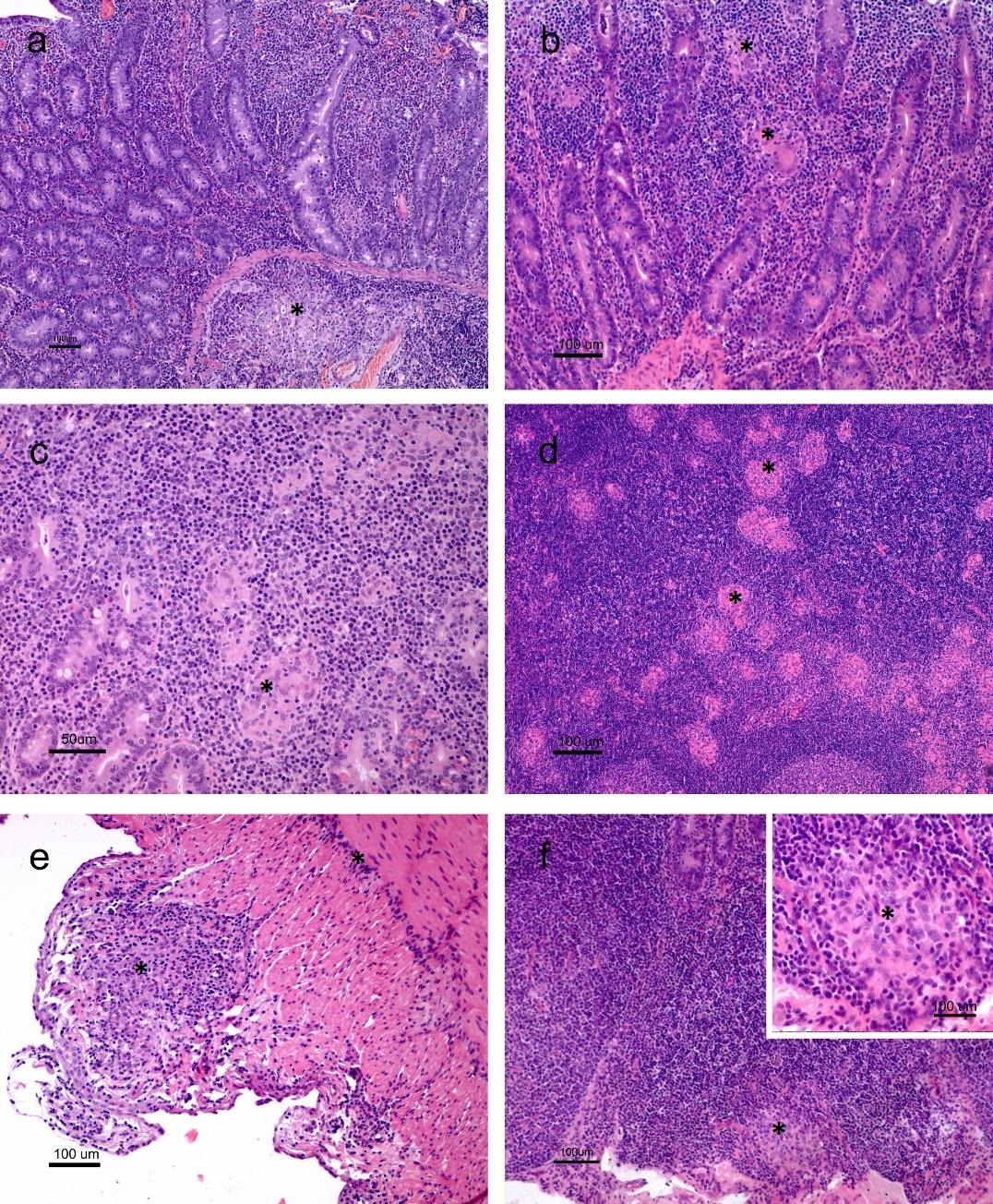


**Supplementary Figure 2**. 330dpi (a) Multifocal lesion a of an animal from NVI group. Vast granulomas in the tissue invading the adjacent lamina propria. Jejunum. HE. (b) and (c) Multifocal lesion b of the NVI group. Granulomas located in the lamina propria not adjacent to lymphoid tissue. Jejunum. HE. (d) Multifocal lesion in lymph node with multiple granulomas in its cortex. HE. (e) Granulomatous serositis in the jejunal serosa of the NVI group. HE. (f) Focal lesion in jejunum with connective tissue in two animals from VI group. Inset: Focal lesion. Small, well-defined granulomas, as can be seen at higher magnifications in group VI. Lesions are indicated with an asterisk.


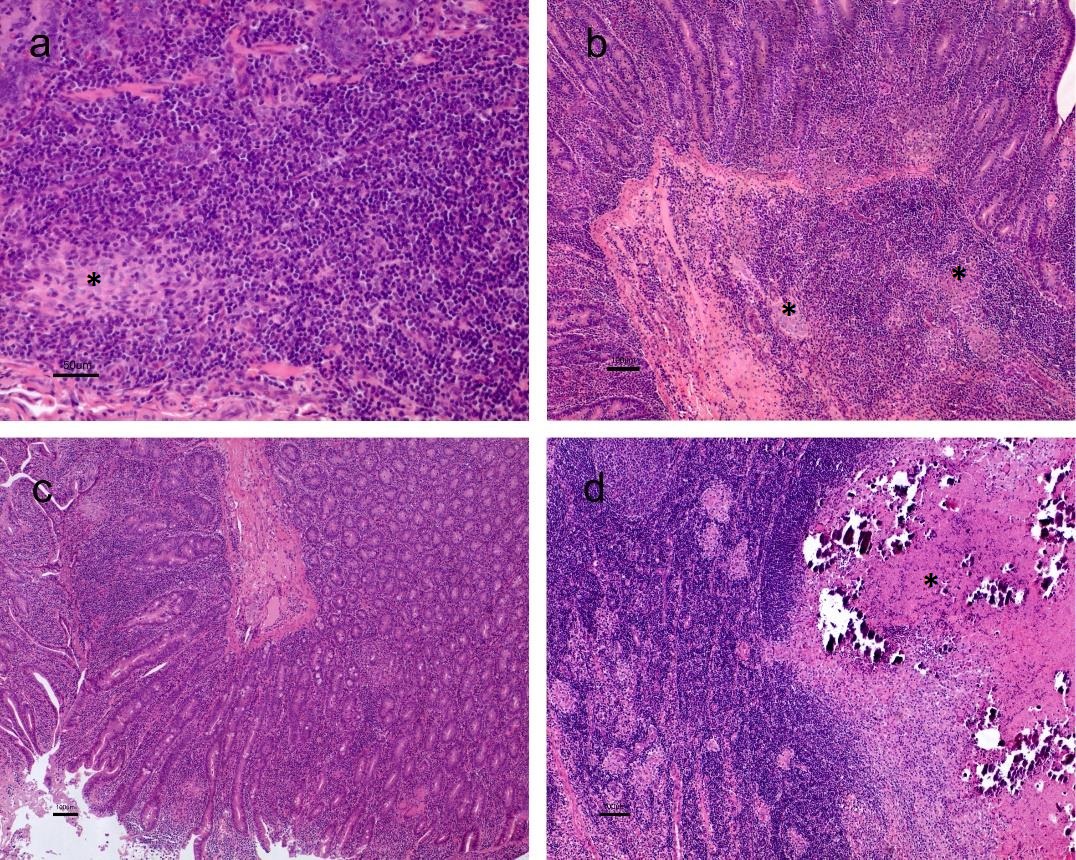


**Supplementary Figure 3.** Lesions found in IV group. 330 dpi Jejunum. HE. (a) Focal lesion in an animal from VI group. Poorly defined and infiltrative granuloma located in the interfollicular area of a Peyer's patch of the jejunum (asterisk). HE. (b) Multifocal lesion type a. Small number of granulomas invade the lamina propria related to the lymphoid tissue (asterisk). Jejunum HE. (c) Multifocal lesion b. Granulomas located in the lamina propria not adjacent to lymphoid tissue and (d) areas of necrosis and mineralization in cortex of villi (asterisk).

| Animal  ID | Exp. group | Slaughter dpi | Culture | | Nested PCR | Lesion type |
| --- | --- | --- | --- | --- | --- | --- |
|  |  |  | **Feces** | **Tissue** |  |  |
| 16 | VI | 120 | - | - | 1/3 | Multifocal a |
| 20 | VI | 120 | - | - | 1/3 | Focal |
| 21 | VI | 120 | - | - | 0/3 | No lesion |
| 25 | VNI | 120 | - | - | 0/3 | No lesion |
| 26 | VNI | 120 | - | - | 0/3 | No lesion |
| 2 | NVI | 120 | - | - | 1/3 | Multifocal a |
| 3 | NVI | 120 | - | - | 1/3 | Multifocal b |
| 6 | NVI | 120 | - | - | 0/3 | Focal |
| 7 | NVI | 120 | - | - | 1/3 | Multifocal a |
| 13 | NVI | 120 | - | - | 0/3 | Focal |
| 31 | NVNI | 120 | - | - | 0/3 | No lesion |
| 32 | NVNI | 120 | - | - | 0/3 | No lesion |
| 1 | NVI | A | na | na | na | na |
| 15 | VI | 330 | - | - | 1/3 | Focal |
| 17 | VI | 330 | - | - | 2/3 | Focal |
| 19 | VI | 330 | - | - | 1/3 | No lesion |
| 22 | VI | 330 | - | - | 1/3 | No lesion |
| 23 | VNI | 330 | - | - | 0/3 | No lesion |
| 9 | NVI | 330 | + | - | 2/3 | Multifocal b |
| 11 | NVI | 330 | - | - | 2/3 | Multifocal a |
| 14 | NVI | 330 | - | - | 2/3 | Multifocal b |
| 4 | IV | 330 | - | - | 0/3 | No lesion |
| 5 | IV | 330 | - | **+** | 3/3 | Diffuse |
| 8 | IV | 330 | - | - | 1/3 | Focal |
| 10 | IV | 330 | - | - | 2/3 | Multifocal b |
| 12 | IV | 330 | - | - | 2/3 | Multifocal b |
| 27 | NIV | 330 | - | - | 0/3 | No lesion |
| 28 | NIV | 330 | - | - | 0/3 | No lesion |
| 34 | NVNI | 330 | - | - | 0/3 | No lesion |
| 18 | VI | B | na | na | na | na |
| 24 | VNI | C | na | na | na | na |
| 29 | NVNI | D | na | na | na | na |
| 30 | NVNI | E | na | na | na | na |

**Supplementary Table 1**. Summary of studies on fecal and tissue cultures, nested PCR, and type of lesion at 120 dpi and 330 dpi in kids from the different studied groups.-: negative; +: positive; nested PCR: number of positive samples/total analyzed; Exp. group: experimental group; VI: vaccinated -30 dpi and infected; VNI: vaccinated at -30 dpi but not infected; NVI: not vaccinated but infected; NVNI: not vaccinated and not infected; IV: infected and vaccinated at 150 dpi; NIV: not infected, vaccinated at 150 dpi. dpi: days post-infection; na: not available. A: animal removed from the study at 96 dpi. dpi: days post-vaccination; B: kid removed at 245 dpi; C: kid removed from the study at 186 dpi; D: kid removed at 238 dpi; E: kid removed at 302 dpi.
